# A self-amplifying USP14-TAZ loop drives the progression and liver metastasis of pancreatic ductal adenocarcinoma

**DOI:** 10.1038/s41418-022-01040-w

**Published:** 2022-07-29

**Authors:** Chunle Zhao, Jun Gong, Yu Bai, Taoyuan Yin, Min Zhou, Shutao Pan, Yuhui Liu, Yang Gao, Zhenxiong Zhang, Yongkang Shi, Feng Zhu, Hang Zhang, Min Wang, Renyi Qin

**Affiliations:** 1 Department of Biliary-Pancreatic Surgery, Affiliated Tongji Hospital, Tongji Medical College, Huazhong University of Science and Technology, 1095 Jiefang Ave, Wuhan, 430030 Hubei China; 2 Hubei Key Laboratory of Hepato-Pancreato-Biliary Diseases, Affiliated Tongji Hospital, Tongji Medical College, Huazhong University of Science and Technology, 1095 Jiefang Ave, Wuhan, 430030 Hubei China

**Keywords:** Oncogenes, Oncogenes

## Abstract

With a 5-year survival rate of approximately 10%, pancreatic ductal adenocarcinoma (PDAC) is one of the most lethal solid malignancies in humans. A poor understanding of the underlying biology has resulted in a lack of effective targeted therapeutic strategies. Tissue microarray and bioinformatics analyses have revealed that the downstream transcriptional coactivator of the Hippo pathway, transcriptional coactivator with PDZ-binding motif (TAZ), might be a therapeutic target in PDAC. Since pharmacological inhibition of TAZ is challenging, we performed unbiased deubiquitinase (DUB) library screening to explore the pivotal regulators of TAZ ubiquitination as potential targets in PDAC models. We found that USP14 contributed to Yes-associated protein (YAP)/TAZ transcriptional activity and stabilized TAZ but not YAP. Mechanistically, USP14 catalyzed the K48-linked deubiquitination of TAZ to promote TAZ stabilization. Moreover, TAZ facilitated the transcription of USP14 by binding to the TEA domain transcription factor (TEAD) 1/4 response element in the promoter of USP14. USP14 was found to modulate the expression of TAZ downstream target genes through a feedback mechanism and ultimately promoted cancer progression and liver metastasis in PDAC models in vitro and in vivo. In addition, depletion of USP14 led to proteasome-dependent degradation of TAZ and ultimately arrested PDAC tumour growth and liver metastasis. A strong positive correlation between USP14 and TAZ expression was also detected in PDAC patients. The small molecule inhibitor of USP14 catalytic activity, IU1, inhibited the development of PDAC in subcutaneous xenograft and liver metastasis models. Overall, our data strongly suggested that the self-amplifying USP14-TAZ loop was a previously unrecognized mechanism causing upregulated TAZ expression, and identified USP14 as a viable therapeutic target in PDAC.

## Introduction

Pancreatic ductal adenocarcinoma (PDAC) is one of the most lethal solid malignancies. Despite the improvement in diagnostic and therapeutic strategies for PDAC, the prognosis is still dismally reflected by a 5-year survival rate of approximately 10% [1, 2]. Various factors, such as the lack of reliable biomarkers for early detection and the limitations of therapeutic strategies, have contributed to this situation [3–5]. Although scientific advances have been made, particularly in visualizing the biomolecular landscape in PDAC models [6], the majority of potential “therapeutic targets” are not clinically actionable. Thus, the exploration of novel druggable targets for PDAC intervention is still needed.

The core components of the Hippo pathway are a series of serine/threonine kinases, scaffolding proteins, the transcriptional cofactor Yes-associated protein (YAP) and the PDZ binding motif (TAZ). Mechanistically, the regulatory protein SAV1 and upstream kinases MST1/2 form a complex to phosphorylate and activate LATS1/2, which subsequently causes the phosphorylation, cytoplasmic sequestration, and ubiquitin-proteasome-mediated degradation of YAP and TAZ [7, 8]. Unphosphorylated YAP and TAZ normally translocate to the nucleus, where they interact with the TEAD/TEF family or several other transcription factors to regulate tissue growth and cell differentiation [9, 10]. Recently, extensive evidence has shown that the Hippo pathway is a crucial barrier to tumorigenesis. Inactivation of the Hippo pathway is often positively correlated with poor prognosis in various solid tumours [11, 12], including PDAC [13, 14], which suggests targeting the Hippo pathway as an attractive anticancer therapeutic strategy.

Decades of experience in small molecule drug development indicated that the most attractive targets for small molecule therapeutics were proteins with enzymatic activity, such as kinases [15]. Indeed, 9E1 was designed to inhibit MST activity in cells [16]. However, unlike the inhibition of other oncogenic kinase pathways, the inhibition of kinases in the Hippo pathway commonly caused tumorigenesis. As the final downstream mediators and key oncoproteins, YAP/TAZ have become the most attractive therapeutic targets in the Hippo pathway [17]. However, pharmacological inhibition of YAP/TAZ was technically challenging because these proteins lacked known catalytic activity and function [18], which prompted us to seek alternative strategies to indirectly inhibit YAP/TAZ. The stability of TAZ in various cancers is strictly controlled by ubiquitin modification [19–21]. It was intriguing to identify deubiquitinases (DUBs) that could stabilize TAZ protein level in PDAC models. Pharmacological inhibition of these selected druggable deubiquitinating enzymes could be an alternative therapeutic strategy for PDAC.

Here, by unbiased DUB library screening in a PDAC model, we found that USP14, a proteasome-associated deubiquitinating enzyme, contributed to both the transcriptional activity and protein level of TAZ by directly interacting with and deconjugating K48-linked polyubiquitin chains from TAZ. We also revealed that USP14 was positively regulated by TAZ-TEAD1/4, thus forming a self-amplifying loop. Gain- and loss-of-function studies indicated that USP14 functioned as an oncogene in PDAC. Moreover, analysis of clinical data from PDAC patients revealed that the protein levels of USP14 and TAZ were positively correlated and that high expression of USP14 was associated with a poorer prognosis in PDAC patients. Furthermore, we introduced a small molecule inhibitor of USP14 catalytic activity, IU1 [22], which inhibited the development of PDAC in subcutaneous xenograft and liver metastasis models. This study also showed that IU1 was a possible lead compound for developing anticancer drugs that targeted tumours with TAZ hyperactivation.

## Results

### Identification of USP14 as a key regulator of Hippo-TAZ signalling in PDAC

**Fig. 1:**
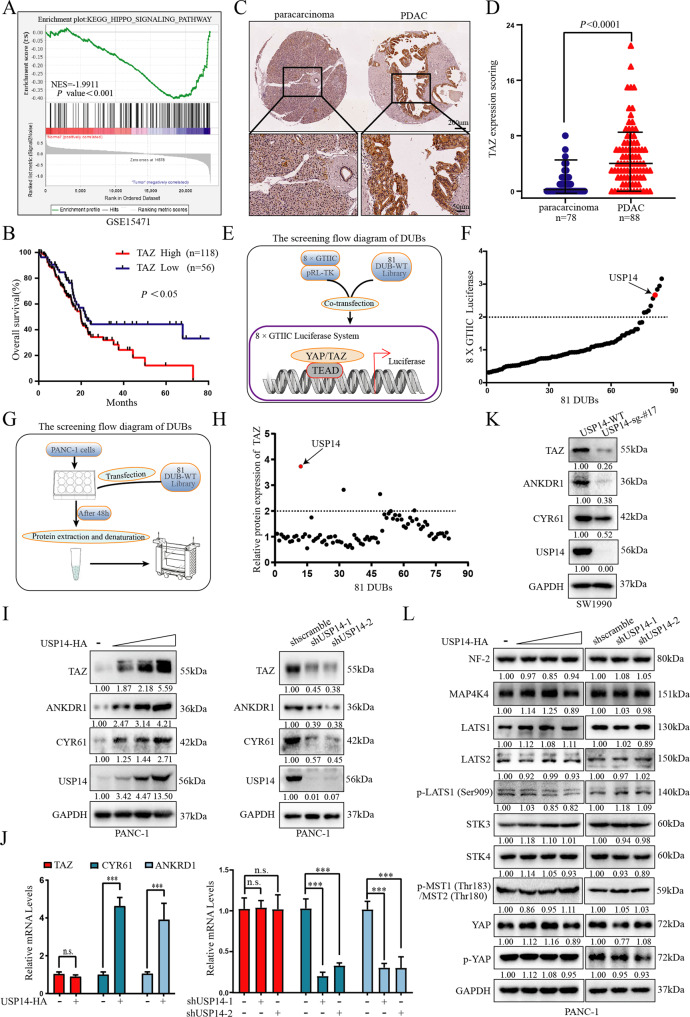
USP14 was identified as a novel regulator of Hippo-TAZ signalling in a PDAC model. **A** GSEA demonstrated that Hippo pathway signalling was significantly changed in PDAC tumour tissue compared with paracarcinoma tissue in a GEO dataset (GSE15471). **B** Individuals in the TCGA-PAAD dataset were divided into the TAZ^high^ expression and TAZ^low^ expression groups according to the expression of TAZ. The overall survival rate of the PDAC patients was estimated by Kaplan-Meier analysis. *P* < 0.05 was considered statistically significant. **C** Immunohistochemistry staining of TAZ in human PDAC TMAs (refer to Fig. S2A); representative images were presented. D. The expression level of TAZ in PDAC and nontumour tissue based on our in-house-constructed human PDAC TMAs. **E** The flow chart for screening DUB(s) that regulated YAP/TAZ activity with the 8 × GTIIC-luciferase system. **F** PANC-1 cells were cotransfected with the 8 × GTIIC-luciferase plasmid, pRL-TK and separate expression plasmids for 81 DUBs. Luciferase reporter assays were performed to screen the DUB(s). *Renilla* luciferase activity was used as the internal reference. Data were representative of three independent experiments with similar results, and each experiment consisted of three repeated biological samples. **G** The flow chart for screening DUBs that regulated TAZ by immunoblotting analysis. **H** The relative expression level of TAZ was calculated by densitometry and plotted. GAPDH was used as the internal reference, and immunoblotting results were shown in Fig. S2C. Data were representative of three independent experiments with similar results. **I**, **L** Immunoblotting analysis were performed to examine the protein levels of core components of the Hippo pathway in the PANC-1 cells transfected with the USP14-HA or USP14 shRNA plasmids. Data were representative of three independent experiments with similar results. **J** RT-qPCR analysis was performed to measure mRNA expression levels in the PANC-1 cells transfected with the USP14-HA or shRNA plasmids. **K** Immunoblot analysis was performed to examine the protein levels of core components of the Hippo pathway in the USP14 knockout SW-1990 cells. See also Figs. S1–3.

Gene set enrichment analysis (GSEA) of Gene Expression Omnibus (GEO) datasets showed that the Hippo pathway was dysregulated in PDAC, and was closely correlated with the initiation and progression of PDAC (Fig. 1A and S1A, B). Furthermore, the GEO database showed that TAZ/YAP expression was significantly upregulated in the PDAC tissues compared to the adjacent tissues (Fig. S1C–H). In addition, Kaplan-Meier survival analysis showed that the TCGA-PAAD patients with the higher TAZ/YAP mRNA expression levels had decreased overall survival(OS) rates and a poorer prognosis (Fig. 1B and S1I). It has been well-documented that the role of YAP was complex and variable, and YAP simultaneously promoted and inhibited cancer. As the function of TAZ was more limited than that of YAP[23–25], we mainly focused on TAZ function. The analysis of human PDAC tissue microarrays (TMAs) revealed that the expression of TAZ was much higher in the PDAC specimens than in the paracarcinoma and normal pancreatic tissues, and that TAZ showed more nuclear accumulation (Fig. 1C, D and S2A). These data suggested that TAZ might be an oncogene and was functionally activated in PDAC cells.

To identify the DUB(s) that potentially regulated TAZ function, we conducted unbiased gain-of-function screening by monitoring 8 × GTIIC-luciferase reporter activity (Fig. 1E). In this screen, 9 DUBs displayed twofold higher YAP/TAZ-TEADs luciferase activity than that of the empty vector (Fig. 1F). In a parallel experiment, we transfected DUBs into PANC-1 cells and monitored the protein level of TAZ (Fig. 1G). The immunoblot results showed that ectopic expression of 3 DUBs caused significant TAZ protein accumulation (Fig. 1H and S2C). Here, we focused on the most promising candidate, USP14, based on these two independent and unbiased screens. Further bioinformatics analysis confirmed the positive correlation of the USP14 mRNA levels with Hippo pathway target genes, including *CYR61, CTGF, ANKRD1*, and *BIRC5* (Fig. S2D–G). Consistent with this finding, knockdown of USP14 reduced the protein levels of TAZ and TAZ target genes in PDAC cells (Fig. 1I, J and S3A, B). A similar effect was confirmed in the cells with CRISPR-mediated USP14 knockout (Fig. 1K and S3C). However, ectopic expression of USP14 led to the opposite effect (Fig. 1I and S3A). Of note, among all core components of the Hippo pathway (NF-2, MAP4K4, LATS1/2, STK3/4, and YAP), only TAZ exhibited a change in expression with USP14 modulation (Fig. 1I, L, and S3A, D). In addition, the mRNA level of TAZ was not significantly altered when USP14 expression was changed (Fig. 1J, S3B). These data suggested that USP14 regulated the Hippo pathway at the level of TAZ through post-translational modification.

### USP14 interacted with and stabilized TAZ in a deubiquitinase activity-dependent manner

**Fig. 2:**
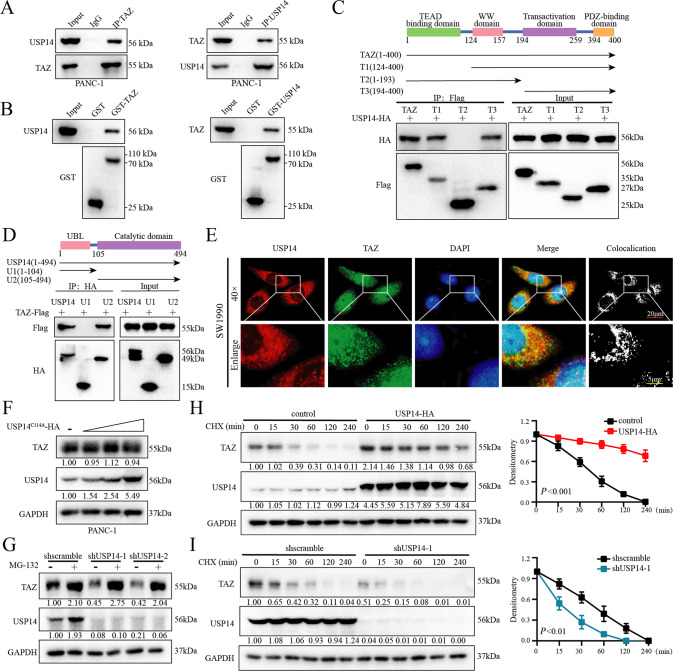
USP14 interacted with and stabilized TAZ in a deubiquitinase activity-dependent manner. **A** Reciprocal Co-IP experiments were performed with the USP14/TAZ antibody in PANC-1 cells. The interaction of endogenous USP14 and endogenous TAZ was analysed by immunoblotting. **B** GST pull-down assay was performed to detect the interaction of USP14 and TAZ. **C**, **D** Plasmids containing full-length and truncated sequences of TAZ and USP14 were constructed, and HEK293T cells were transfected with the indicated plasmids. A Co-IP assay was performed to explore the binding regions between USP14 and TAZ. **E** SW-1990 cells were transfected with the USP14-HA and TAZ-Flag plasmids. After 48 h transfection, an immunofluorescence assay was conducted to acquire the images by using fluorescence microscopy. **F** PANC-1 cells were transfected with the USP14^C114A^-HA plasmid as indicated. Immunoblotting analysis was carried out to examine the protein expression of TAZ and USP14. **G** HEK293T cells were transfected with shScramble or shRNA targeting USP14. After 48 h, these cells were treated with or without MG132 (10 µM) for 6 h. Immunoblotting analysis was used to examine USP14 and TAZ expression. **H**, **I** PANC-1 cells transfected with the indicated plasmid were treated with CHX (50 μM) for the indicated time intervals. Cells were harvested, and the expression of TAZ and USP14 was measured by immunoblotting. The level of protein expression was quantified by densitometry and plotted. GAPDH was used as the internal reference, and the protein expression of TAZ was normalized to that at the *t* = 0 time point. The data were analysed by one-way ANOVA and were presented as the mean ± SD. See also Fig. S4.

To investigate the mechanism of USP14-mediated upregulation of TAZ protein expression, we performed reciprocal coimmunoprecipitation (Co-IP) experiments in HEK293T and PDAC cells. The results showed a physical interaction between the TAZ and USP14 proteins (Fig. 2A and S4A–D). In addition, GST pull-down assay showed that USP14 directly binded to GST-TAZ, and that TAZ directly binded to GST-USP14 but not GST (Fig.2B). In addition, the domain between amino acids (aa) 194-400 (T3) in TAZ and the domain between aa 105-494 (U2) in USP14 contributed to their interaction (Fig. 2C, D). In addition, the interaction between USP14 and TAZ was further confirmed by immunofluorescence (IF) analysis (Fig. 2E and S4E). Considering that USP14 was mainly expressed in the cytoplasm (Fig. 2E and S4E), the Co-IP experiments were performed in the nuclear and cytoplasmic extract, and demonstrated the interaction between TAZ and USP14 was occurred in the cytoplasm, but not in the nuclear (Fig. S4F).

To investigate whether USP14 binded with TAZ and acted as its DUB in PDAC cells, a USP14 mutant (C114A) [22] was transfected into PDAC cells and found not to affect the protein and mRNA level of TAZ (Figs. 1I, J, 2F, and S3A, S4G, H), indicating that the enzymatic deubiquitination activity of USP14 was required for TAZ stability. Additionally, knockdown of USP14 significantly reduced the TAZ protein level, which was restored in the cells treated with the proteasome inhibitor MG132 (Fig. 2G). Cycloheximide (CHX) chase assay showed that the half-life of the TAZ protein was increased in the USP14 overexpressing cells, while USP14 depletion resulted in accelerated degradation of TAZ (Fig. 2H, I).

### USP14 removed K48-linked polyubiquitin chains from TAZ

**Fig. 3:**
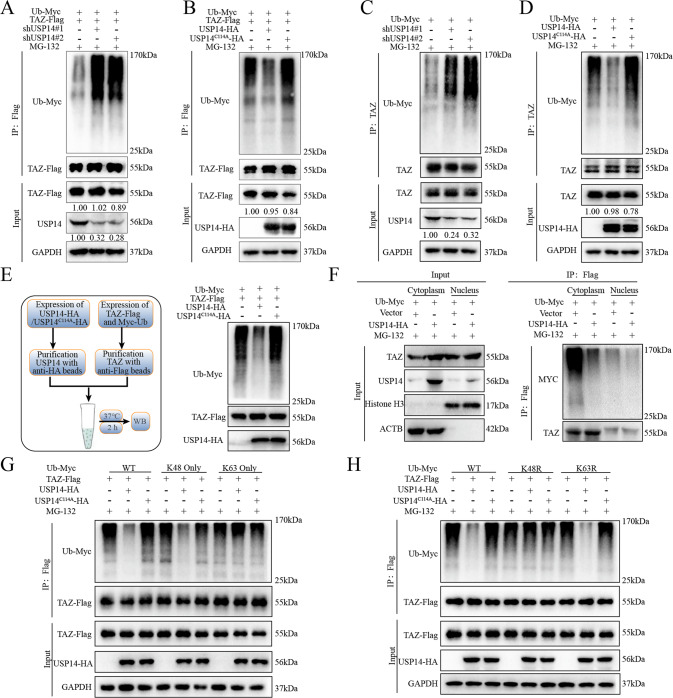
USP14 removed K48-linked polyubiquitin moieties from TAZ. **A**, **B** The ubiquitination assay was performed with exogenously overexpressed TAZ (FLAG-tagged), Ub (MYC-tagged) and shUSP14/USP14-HA /USP14^C114A^ (HA-tagged) in HEK293T cells. After 48 h, the cells were treated with MG132 (10 µM) for 6 h, cell lysates were collected, and immunoprecipitation was performed to analyse the ubiquitination of TAZ. **C**, **D** HEK293T cells were cotransfected with Ub-Myc and shUSP14, and treated with MG132 (10 µM) for 6 h after 48 h of transfection, and immunoprecipitation was performed to analyse the ubiquitination of TAZ. **E** In the in vitro ubiquitination assay, HEK293T cells were transfected with the Ub-Myc and TAZ-Flag plasmids. After 48 h, the cells were treated with MG132 (10 µM) for 6 h and purified with anti-Flag magnetic beads. Then, purified USP14-HA and USP14^C114A^-HA were incubated with purified TAZ protein in deubiquitination buffer for 2 h in a water bath at 37 °C. **F** PANC-1 cells with stable overexpression of TAZ-Flag were separated into the nucleus and cytoplasm by nuclear and cytoplasmic protein extraction kits, Co-IP experiments were conducted, and the stabilization of TAZ in the nucleus or cytoplasm by USP14 was detected. **G**, **H** The ubiquitination assay was performed with exogenously overexpressed TAZ (TAZ-FLAG), Ub (Ub-MYC/K63R-MYC/K48R-MYC/K63O-MYC/K48O-MYC) and USP14 /USP14^C114A^ (HA-tagged) in HEK293T cells. Cells were treated with MG132 (10 µM) for 6 h after 48 h of transfection, and immunoprecipitation was performed to analyse the ubiquitination of TAZ.

Encouraged by these findings, we sought to determine whether USP14-mediated stabilization of the TAZ protein was mediated by deubiquitination. The cell deubiquitination assay was performed to examine the ubiquitination of the TAZ protein. As expected, knockdown of USP14 resulted in increased polyubiquitination of TAZ (Fig. 3A, C), and ectopic expression of USP14 notably reduced the ubiquitination level of exogenous TAZ, whereas the USP14^C114A^ mutant lost the ability (Fig. 3B, D). Then, the in vitro ubiquitination assay demonstrated that purified USP14 decreased the ubiquitination of TAZ but not the USP14^C114A^ mutant (Fig. 3E). Then, we conducted a Co-IP experiment in the nuclear and cytoplasmic protein extraction, and our results demonstrated that USP14 stabilized TAZ in the cytoplasm (Fig. 3F). Previous studies demonstrated that TAZ could be ubiquitinated with lysine 48 (K48)- and K63-linked polyubiquitin chains, both of which promoted proteasome-mediated degradation of TAZ [20, 26]. To identify the type of polyubiquitin chains removed by USP14, we cotransfected TAZ-Flag with Myc-Ub [WT, K48 only (ubiquitin with only the Lys48 residue intact), or K63 only] into HEK293T cells in the presence or absence of USP14-HA/USP14^C114A^-HA. The cell deubiquitination assay results showed that USP14 significantly decreased the K48-linked polyubiquitination of TAZ but had no apparent effect on its K63-linked polyubiquitination (Fig. 3G). Furthermore, the results of the parallel ubiquitination assay with the K48R (mutation of only Lys48 to Arg) mutant showed no increase in TAZ ubiquitination in the presence of USP14 (Fig. 3H). This effect was dependent on the deubiquitination activity of USP14 since the USP14^C114A^ mutant was incapable of hydrolyzing polyubiquitin chains (Fig. 3B, D, E, G, H). Collectively, these results revealed that USP14 deubiquitinated and stabilized TAZ by removing K48-linked polyubiquitin chains from TAZ, thus preventing TAZ proteasomal degradation.

### USP14 promoted pancreatic tumour growth and metastasis

**Fig. 4:**
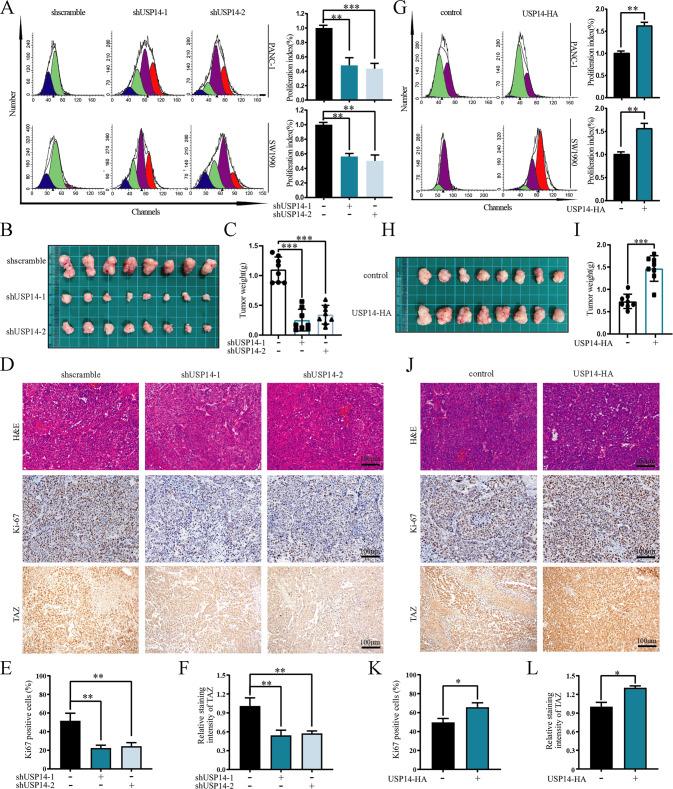
USP14 promoted pancreatic tumour growth. **A**, **G** PANC-1 and SW1990 cells with stable overexpression or knockdown of USP14 were stained with CFSE. After 48 h of staining, flow cytometry was used to examine the proliferation of cells. The experiment was technically repeated three times. **B**, **H** SW1990 cells with stable knockdown of USP14 were implanted subcutaneously into the right axillae of nude mice. After 33 days or 27 days, ectopic xenograft tumours were excised and photographed (*n* = 8). **C,** **I** Tumour weight was shown related to **B** and **H** (*n* = 8). **D**–**F**, **J**–**L** IHC staining of H&E, Ki67 and TAZ was shown. The percentage of Ki67-positive cells and the relative staining intensity of TAZ were quantified (*n* = 8). See also Fig. S5.

Given the important role of USP14 in regulating TAZ protein stability and the function of the Hippo pathway and TAZ in tumorigenesis, we next assessed the biological role of USP14 in PDAC. Cell Counting Kit-8 (CCK-8) and carboxyfluorescein diacetate succinimidyl ester (CFSE) labelling with flow cytometry analysis were performed to examine cell proliferation, which showed that USP14 depletion significantly decreased the proliferation of PDAC cells, whereas ectopic expression of USP14 led to the opposite phenotype (Fig. 4A, G and S5A–D). Flow cytometry analysis was used to examine the cell cycle and apoptosis, and the results showed that knockdown of USP14 significantly inhibited the G1/S phase transition in PDAC cells (Fig. S5E, F), and increased apoptosis (Fig. S5G, H). Next, subcutaneous xenograft models showed that USP14 deficiency significantly inhibited the growth of tumours, and decreased the expression of TAZ and Ki-67 (Fig. 4B–F and S5I). Conversely, overexpression of USP14 had the opposite effect (Fig. 4H–L and S5J). These data highlighted the role of USP14 in promoting PDAC tumour growth and cell proliferation.

**Fig. 5:**
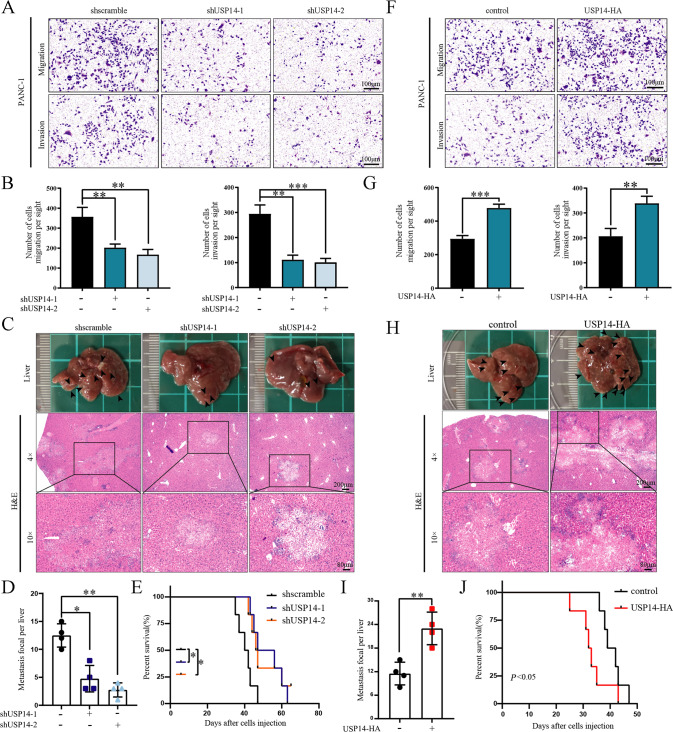
USP14 promoted pancreatic cancer liver metastasis. **A**, **F** Transwell assays were used to measure the migration and invasion of PANC-1 cells with USP14 overexpression or knockdown. Representative images were presented. The experiment was technically and biologically repeated three times. **B**, **G** Cells on the bottom surface of the chamber membrane were counted. **C**, **H** SW1990 cells with stable overexpression or knockdown of USP14 were injected into the spleens of nude mice. After 30 days, the livers were excised and photographed. A representative liver was shown. The black arrows indicated liver metastatic nodules. H&E staining showed liver metastatic nodules (*n* = 10). **D**, **I** The liver metastatic nodules were counted. and the data were analysed by Student’s *t-test* (*n* = 4). **E**, **J** Kaplan-Meier survival analysis was performed to evaluate the overall survival of mice (*n* = 6). See also Fig. S6.

Then, we evaluated the effects of USP14 on the invasion and metastasis of PDAC. Transwell assays and wound healing assays showed that the USP14 depletion reduced the migration and invasion of PDAC cells, whereas USP14 overexpression enhanced these abilities (Fig. 5A, B, F, G and S6A–D). Nextly, we established a liver metastasis model by hemi-spleen injection liver metastasis model by intrasplenic injection to evaluate the influence of USP14 on PDAC liver metastasis in vivo. The results showed that depletion of USP14 decreased the number of micrometastases and increased the OS of mouse, while overexpression of USP14 led to a significant increase in liver metastasis and reduced the OS time of the mice (Fig. 5C–E, H–J). In addition, the performed H&E staining of the lungs showed that there were no obvious lung metastases (Fig. S6E, F). Together, these data indicated that USP14 played a prometastatic role in PDAC liver metastasis.

### USP14 promoted PDAC progression in a TAZ-dependent manner

**Fig. 6:**
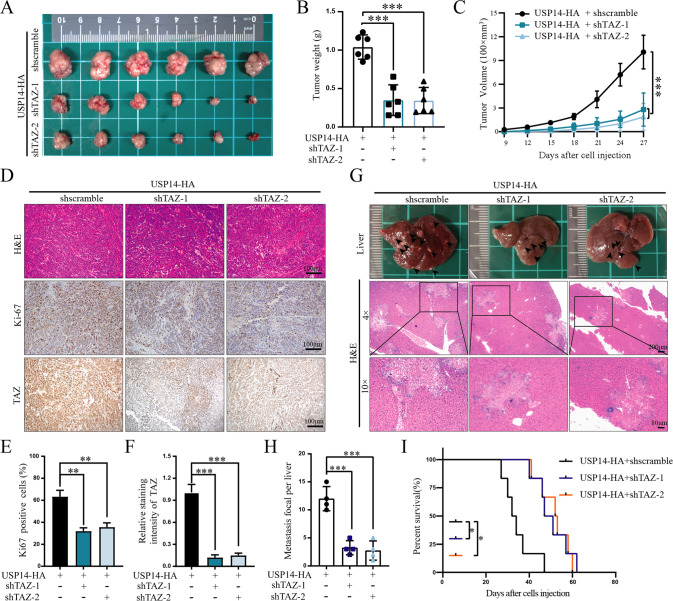
USP14 promoted pancreatic cancer progression in a TAZ signalling-dependent manner. **A** SW1990 cells with stable overexpression of USP14 and knockdown of TAZ were implanted subcutaneously into the right axillae of nude mice. After 30 days, ectopic xenograft tumours were excised and photographed (*n* = 6). **B** Tumour weights were shown (*n* = 6). **C** Tumour volumes were measured at 2-day intervals, and growth curves of xenograft tumours were presented. The data were analysed by one-way ANOVA and were presented as the mean ± SD values (*n* = 6). **D** H&E staining and IHC (Ki-67 and TAZ) were performed on xenograft tumours originating from SW1990 cells with stable overexpression of USP14 and knockdown of TAZ. Representative IHC image s were shown (*n* = 6). **E**, **F** The percentage of Ki67-positive cells and the relative staining intensity of TAZ were quantified. Scale bar, 100 µm (*n* = 6). **G** SW1990 cells with stable overexpression of USP14 and knockdown of TAZ were injected into the spleens of nude mice. After 30 days, the livers of the mice were excised and photographed, and a representative liver was shown. The black arrows indicated liver metastatic nodules. H&E staining showed liver metastatic nodules (*n* = 10). **H** The number of liver metastatic nodules was determined and analysed by Student’s *t*-test (*n* = 4). **I** Kaplan-Meier survival analysis was performed to evaluate the OS of mice (*n* = 6).

We next investigated whether USP14-mediated TAZ stabilization is required for the regulatory effect of USP14 on cell proliferation and metastasis in PDAC. TAZ was knocked down in PDAC cells with USP14 overexpression (Fig. S7A, B). CFSE labelling with flow cytometry analysis and tranwell assay showed that TAZ inhibition significantly blocked USP14-induced cell proliferation, migration, and invasion (Fig. S7C, D). Subcutaneous xenograft and liver metastasis models were established to further confirm the roles of TAZ in USP14-mediated tumour progression in vivo. It observed that knockdown of TAZ decreased the growth of the USP14-overexpressing tumours, and inhibited the expression of Ki67 and TAZ (Fig. 6A–F). In the liver metastasis model, the number of liver micrometastases was significantly decreased, and the OS of TAZ depletion mice was increased, compared with control group (Fig. 6G–I). Taken together, these results indicated that USP14 promoted PDAC proliferation and metastasis in a TAZ signalling-dependent manner.

### Aberrant expression of USP14 in PDAC samples

The expression profile of TAZ in PDAC samples (Fig. S2A) and the correlation between USP14 and TAZ expression prompted us to explore the importance of USP14 expression in patients with PDAC. USP14 expression was significantly upregulated in the PDAC tissues compared to the adjacent tissues in the GEO database (Fig. S8A–C). Kaplan-Meier survival analysis showed that the PDAC patients with higher USP14 mRNA expression levels had decreased OS in the TCGA-PAAD dataset (Fig. S8D). A total of 18 pairs of fresh matched PDAC surgical tissues were collected and subjected to analysis of USP14 protein and mRNA levels. As shown in Fig.S8E, F, the USP14 level was significantly higher in the PDAC samples than in the paired adjacent tissue samples. In addition, the protein level of USP14 was higher in PDAC cells than in immortalized HPDE cells (Fig. 8G). Consistented with these findings, the USP14 level was increased and had a significant positive correlation with TAZ in TMAs (Fig. S2A, B, S8H–J). Univariate and multivariate analyses were performed according to USP14 expression and clinical information in our human PDAC TMAs, revealing that like clinical stage and T classification, the USP14 expression was also an independent predictor of OS (Tables S1–4). Thus, these data confirmed the pivotal role of USP14 in the progression and prognosis of human PDAC.

### TAZ positively regulated USP14 expression at the transcriptional level

**Fig. 7:**
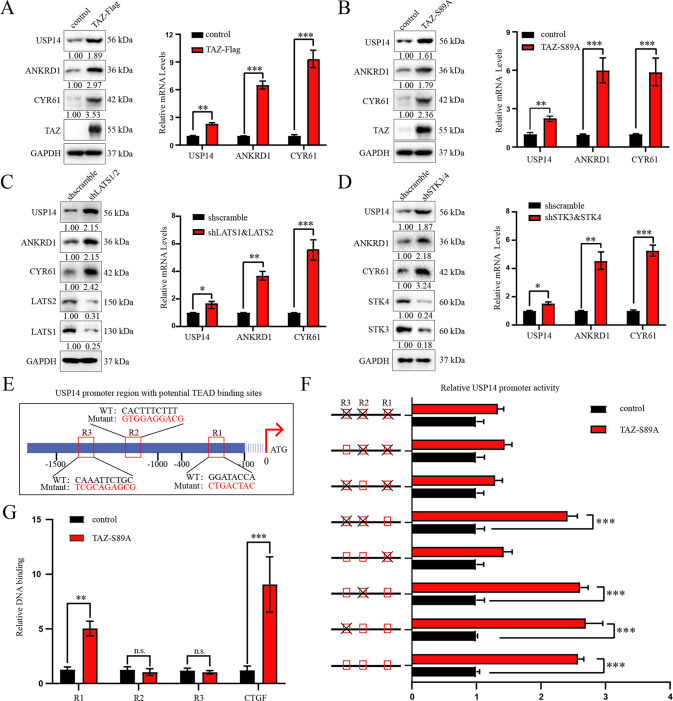
TAZ positively regulated USP14 expression at the transcriptional level. **A**, **B** SW1990 cells were transfected with the TAZ-Flag (2.0 μg plasmid according to per well of 6-well plate) and TAZ-S89A plasmids (1.0 μg plasmid according to per well of 6-well plate), immunoblotting and RT-qPCR were used to measure the protein and mRNA expression levels of USP14 and TAZ target genes. **C**, **D** SW-1990 cells were transfected with the shLATS1/2 (LATS1 and LATS2 double knockdown) or shSTK3/4 (STK3 and STK4 double knockdown) plasmids, and immunoblotting and RT-qPCR were used to measure the protein and mRNA expression levels of USP14 and TAZ target genes. **E** A schematic diagram of the USP14 promoter region with potential TEAD1/4 binding sites (TBSs) was shown. The wild-type and TBS mutant sequences were indicated. R1, R2, and R3 indicate three sites (Region 1, Region 2, and Region 3) in the USP14 promoter containing the potential TBSs. **F** The luciferase reporter assay showed that TAZ promoted the expression of USP14 by affecting the USP14 promoter region (R1). *Renilla* luciferase activity was used as the internal reference. Data were representative of three independent experiments with similar results, and each experiment consisted of three repeated biological samples. **G** A ChIP-qPCR assay was used to examine the binding of TAZ and the USP14 promoter in TAZ^S89A^-Flag overexpressing PANC-1 cells. CTGF was used as a positive control. Data were representative of three independent experiments with similar results.

In the rescue experiment, we unexpectedly observed that TAZ depletion reduced the protein and mRNA expression levels of USP14 (Fig. S7A, B). This finding prompted us to examine whether the USP14 gene was potentially regulated by TAZ. Surprisingly, both the mRNA and protein levels of USP14 were significantly increased in the TAZ-Flag-overexpressing and constitutively active form of TAZ (TAZ-S89A) cells (Fig.7A, B). Since TAZ was negatively regulated by LATS1/2 and STK3/4, we constructed cell lines with double knockdown of LATS1/2 (shLATS1/2) or shSTK3/4. The results showed that the inactivation of Hippo pathway enhanced the USP14 abundance at both the mRNA and protein levels (Fig. 7C, D). In addition, knockdown of YAP could inhibit the protein and mRNA levels of USP14 while overexpression of YAP had the opposite effect in PDAC cells (Fig. S9A–D).

Due to the lack of DNA binding domains, TAZ commonly serves as a transcriptional coactivator and has been reported to interact with multiple transcription factors, especially TEAD transcription factors[8]. Thus, we screened the USP14 promoter region for possible TEAD binding sites with the JASPAR (http://jaspardev.genereg.net/) database and found 3 conserved TEAD binding sites (Fig. 7E). Dual-luciferase reporter assays showed the R1 (binding region 1) mutation almost completely abolished the promoter inducibility. In addition, the ChIP-qPCR experiment demonstrated that the TAZ could bind to the USP14 promoter (Region 1) at the chromatin level (Fig. 7G). Collectively, these data indicated that USP14 was regulated by TAZ at the transcriptional level.

### Therapeutic effects of a USP14 inhibitor

**Fig. 8:**
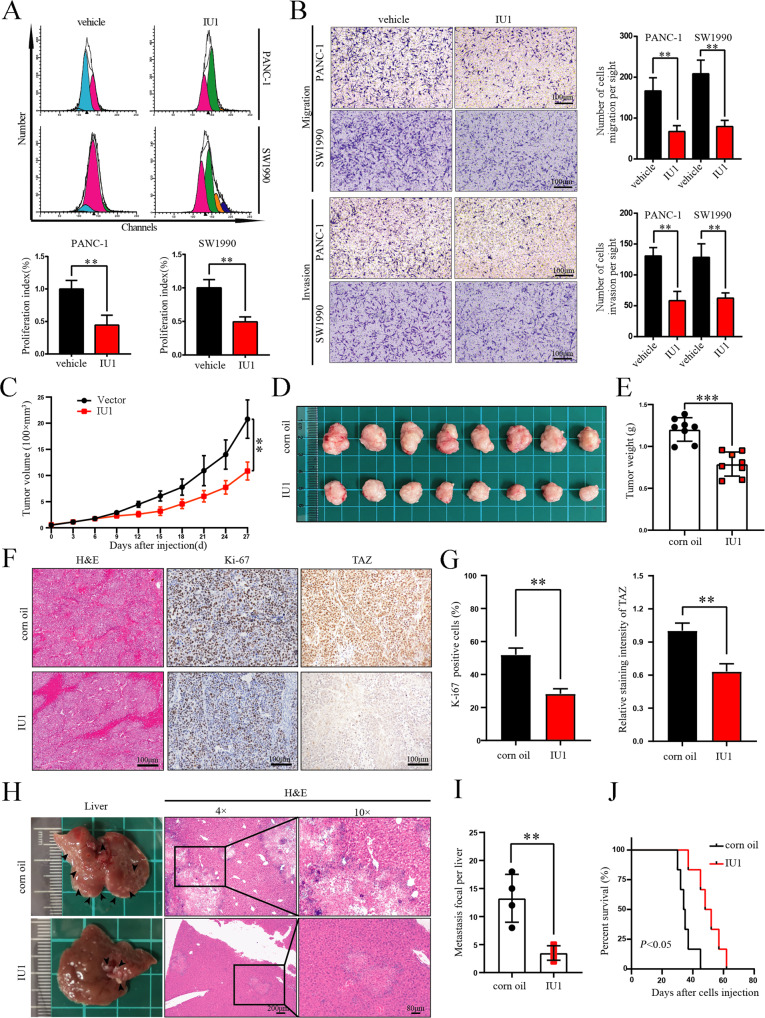
Therapeutic effects of a USP14 inhibitor. **A** PANC-1 and SW1990 cells were stained with CFSE and plated into 6-well plates for 12 h of incubation. After 48 h IU1 treatment, flow cytometry was used to examine the cell proliferation. **B** Transwell assay was used to measure the migration and invasion of the PANC-1 and SW-1990 cells treated with IU1. **C** Tumour volumes were measured at 2-day intervals. The data were analysed by one-way ANOVA and were presented as the mean ± SD (*n* = 8). **D** Subcutaneous tumours from the vehicle-treated group and the IU1-treated group were shown (*n* = 8). **E** Tumour weights referred to in Fig. 8**D** were shown (*n* = 8). **F**, **G** H&E staining and IHC staining of Ki67 and TAZ were shown. The percentage of Ki67-positive cells and the relative staining intensity of TAZ were quantified (*n* = 8). **H** Representative livers from the corn oil-treated group and the IU1-treated group were shown. **I** The number of liver metastatic nodules was determined and plotted (*n* = 4). **J** Kaplan-Meier survival analysis was performed to evaluate the OS of mice (*n* = 4).

After elucidating the function of USP14 in PDAC progression and liver metastasis and clarifying its underlying mechanisms, we next evaluated the potential therapeutic effect of the USP14-specific inhibitor IU1 in PDAC models. CFSE labelling with flow cytometry analysis and transwell assays showed that IU1 significantly decreased the proliferation index, invasion and metastatic abilities of PDAC cells (Fig. 8A, B). Subcutaneous xenograft and liver metastasis models were also used to examine the therapeutic effects of IU1 in vivo. Notably, IU1 administration inhibited the tumour growth and reduced the expression of TAZ and Ki-67 (Fig. 8C–G). It was worth noting that IU1 administration reduced liver micrometastases and improved OS (Fig. 8H–J). These findings clearly indicated that IU1 treatment could prevent PDAC progression and liver metastasis in mouse models.

## Discussion

PDAC was considered the third leading cause of cancer-related mortality [1]. Although considerable efforts have been made to visualize the biomolecular landscape and exploit novel targeted therapeutic regimens in recent decades [27, 28] identification of novel therapeutic targets was still needed. The Hippo pathway was tightly involved in numerous human cancers, and targeting components of this pathway was a new strategy for cancer treatment [9, 11, 29]. However, the development of small molecule drugs that targeted the Hippo pathway for cancer treatment remained technically challenging. Thus, we focused on DUBs, which positively regulated their substrates, as pharmacological targeting of DUBs could be an alternative strategy to address the undruggability of their substrates. In this study, we verified that TAZ was functionally activated and functioned as an oncogene in PDAC. By unbiased gain-of-function screening, we identified USP14 as the most potent DUB that regulated TAZ ubiquitination and stability. In vitro and in vivo functional experiments confirmed that USP14 was a driver of cancer progression and liver metastasis in PDAC. Moreover, TAZ/TEADs positively regulated USP14 expression at the transcriptional level, indicating that USP14 and TAZ formed a positive feedback loop to promote PDAC development. Additionally, we found that administration of a USP14-specific inhibitor, IU1, prevented PDAC progression and liver metastasis in mouse models. Notably, USP14 was related to the proteasome and played an important role in regulating protein degradation [30, 31]. Our results demonstrated that USP14 binded TAZ, and regulated TAZ through posttranslational modification. However, USP14, as a proteasome-associated DUB, also targeted many other substrates in a relatively nonspecific manner. The mechanism of USP14 should be further explored.

As described earlier, USP14 depletion accelerated K48-linked ubiquitination and reduced the stability of the AR protein, which further suppressed the proliferation of AR-responsive breast cancer cells [32]. In addition, USP14 was reprorted to be ectopically expressed in various cancer types, including bladder cancer, lung cancer, breast cancer, and PDAC [33]. More recently, Hang et al. reported that USP14 promoted cell proliferation, invasion, and migration and prevented apoptosis in vitro [34]. However, whether USP14 contributed to PDAC progression and metastasis in animal models and the identity of the precise mechanism remained unknown. Consistent with the above findings, we showed that USP14 deubiquitinated and stabilized TAZ, which further promoted PDAC progression and liver metastasis in vivo in a TAZ-dependent manner. This study supported the previous observations that USP14 could promote the progression of PDAC.

YAP/TAZ are considered the major downstream effectors of the Hippo pathway, and their functions were regulated by different PTMs, such as phosphorylation and ubiquitination [35, 36]. Since the E3 ubiquitin ligase SKP1–cullin–F-box (SCF) regulated YAP/TAZ ubiquitination and consequent degradation [37, 38], it has been extensively studied with a focus on identifying the dubs that regulated YAP/TAZ deubiquitination. Indeed, USP10 and OTUB2 were recently reported as the candidate proteins that deubiquitinated and stabilized YAP/TAZ in the liver and breast cancer models, respectively [19, 21]. Additionally, evidence showed that USP1 functions as a deubiquitinase to regulate TAZ specifically in breast cancer[39]. However, none of the abovementioned DUBs simultaneously affected the protein level and transcriptional activity of TAZ in our screening systems. Several factors may contribute to these seemingly contradictory results. Firstly, the candidate DUBs identified in the initial screening step were limited by the differences in the DUB library constituents. This discrepancy may also arise from the specific cell lines used for screening or from the overexpression/knockdown efficiency of each DUB in different studies. After USP14 was identified, we further examined the levels of core components in the Hippo pathway to confirm that USP14 regulated the Hippo pathway at the level of TAZ. Then, we mainly focused on the molecular mechanism by which USP14 regulates TAZ and highlighted the antitumour effect of IU1 in PDAC mouse models. However, given the limitation of this screening approach, we should not ignore the role of other DUBs in PDAC.

The overlapping and divergent functions of YAP and TAZ were supported by the finding that YAP knockout was embryonically lethal in mice, while TAZ knockout mice developed only kidney and lung disease[40–42]. YAP and TAZ also played diverse roles in promoting malignant transformation and tumour progression [43, 44], a property that could be attributed to their drivers and the nonoverlapping transcriptional programs controlled by them [45]. YAP has also been reported as a tumour suppressor in some cancers, including colorectal cancer and ERα-positive breast cancer [46]. Pearson et al. reported that YAP had opposite pro- or anticancer activity in cancers with different YAP/TAZ expression backgrounds [47]. Our data intensively proved that of all the core components of the Hippo pathway, USP14 only regulated TAZ protein levels and did not alter YAP protein levels. This discovery was conducive to novel opportunities and strategies for potential TAZ-targeted cancer therapy.

Taken together, via unbiased gain-of-function DUB screening, we showed that USP14 acted as a positive regulator of TAZ by removing K48-linked polyubiquitin chains from TAZ, whereas TAZ promoted USP14 transcriptional activity. USP14 and TAZ formed a positive feedback loop to promote PDAC progression and liver metastasis. The USP14 inhibitor IU1 may be a possible lead compound for developing anticancer drugs for the treatment of PDAC. In subsequent studies, there are still pressing needs in exploring the biological security and optimizing the structural features of IU1 for combating PDAC.

## Materials and methods

### Patient samples

Human pancreatic tissue samples were collected from individuals who were diagnosed with pancreatic cancer by pathological examination and underwent surgical resection in the Department of Biliary-Pancreatic Surgery at Tongji Hospital of Huazhong University of Science and Technology (Wuhan, China). Follow-up of the patients was performed after surgery, and the date of death or last follow-up was recorded. The procedure for patient tissue sample collection was approved by the Ethics Committee of Tongji Hospital, Huazhong University of Science and Technology, and abided by the principles of the Declaration of Helsinki.

### Cell culture

Human embryonic kidney (HEK293T) cells, immortalized human pancreatic ductal epithelial (HPDE) cells and human pancreatic cancer cells (PANC-1, SW1990, Panc03.27, BxPC-3, AsPC-1, MIA Paca-2, and CFPAC-1) (CTCC, Hangzhou, China) were cultured in DMEM or RPMI 1640 medium (Solarbio Science & Technology Co., Beijing, China) with 10% fetal bovine serum (FBS, Cegrogen, Stadtallendorf, Germany), 100 units/ml penicillin and 100 μg/ml streptomycin at 37 °C in a humidified atmosphere containing 5% CO_2_. All of the cell lines were examined for short tandem repeat (STR) and mycoplasma infection periodically.

### Luciferase reporter assay

The 8 × GTIIC-luciferase reporter vector, which contains eight TEAD binding sites, can be used to monitor the transcriptional activity of YAP/TAZ. The USP14 promoter fragment (−1410 bp–168 bp) was synthesized (Tsingke, Wuhan, China) and inserted into the pGL3-Basic vector. Potential TEAD1/4 binding sites in the USP14 promoter region were predicted with the JASPAR website (http://jaspar.genereg.net/), and the binding sites were mutated by inverse PCR. HEK293T cells were cotransfected with the indicated vector and pRL-TK. After 48 h, a Dual-Luciferase Reporter Assay Kit (Vazyme, China) was used to measure firefly luciferase activity, and *Renilla* luciferase activity was used as the internal reference.

### Co-IP

Cells were transfected with the indicated plasmids or subjected to the indicated treatment for 24 h and were then collected and lysed in 1 ml of IP buffer [20 mM Tris-HCl (pH 7.4), 150 mM NaCl, 1 mM EDTA, and 1% NP-40] with complete protease inhibitor cocktail (Ref: 001,04,132,693, Roche, Indianapolis, IN) and PhosSTOP (Ref: 001,04,837,906, Roche, Indianapolis, IN). The cell lysate was subjected to ultrasonication and centrifugation at 12,000 rpm for 15 min at 4 °C. Fifty microlitres of lysate supernatant were taken as input, and the rest was incubated overnight with Protein A/G Magnetic Beads (B23202, Biomake, US) and the indicated antibody at 4 °C on a rotating device. Next, the magnetic beads were washed 4 times for 5 min each with cold IP buffer and boiled for 15 min in 30 µl of 2 × loading buffer. After centrifugation, the supernatant was collected, and immunoblotting analysis was performed. The antibodies are listed in Table S5. The full-length uncropped original western blots used in their manuscript are uploaded in Supplemental Material.

### Ubiquitination assays

The pRK5-HA-ubiquitin-K48 plasmid and pRK5-HA-ubiquitin-K63 plasmid were purchased from Addgene. Then the ubiquitin coding sequence was amplified and subcloned into the pcDNA3 1-Myc plasmid. For the cell ubiquitination assay, cells were transfected with the Ub-Myc and TAZ-Flag plasmids with or without USP14-HA or USP14^C114A^-HA. After 36 h, the cells were treated with MG132 for 6 h and collected with 50 μl of SDS lysis buffer [20 mM Tris-HCl (pH 7.4), 150 mM NaCl, 1 mM EDTA, and 1% SDS]. The lysates were subjected to heating at 99 °C for 10 min and diluted with 500 μl of IP buffer. The next steps were the same as those used for Co-IP. For the in vitro ubiquitination assay, HEK293T cells were transfected with the Ub-Myc and TAZ-Flag plasmids. After 48 h, the cells were treated with MG132 (10 µM) for 6 h and purified with anti-Flag magnetic beads. Then, purified USP14-HA and USP14^C114A^-HA were incubated with purified TAZ protein in deubiquitination buffer (20 mM Tris-Cl, 5 mM MgCl_2_, 2 mM dithiothreitol, pH 7.5,10 mM ATP) for 2 h in a water bath at 37 °C.

### Total RNA isolation and reverse transcription real-time PCR

Cells were treated as indicated. Total RNA was extracted with RNA Isolater Total RNA Extraction Reagent (TRIzol, Vazyme, Jiangsu, China), and cDNA was generated using HiScript® III RT SuperMix for qPCR (Vazyme) according to the manufacturer’s protocol. ChamQ Universal SYBR qPCR Master Mix (Vazyme) was used for RT-qPCR, ACTB was used as the internal reference, and the results were analysed with Bio-Rad CFX Manager 2.1. The primers are listed in Table S6.

### Plasmid construction, transfection and viral infection

The full-length and truncated sequences of TAZ and USP14 were generated by PCR from human cellular cDNA and cloned into the pHAGE-Flag or pHAGE-HA vector. The TAZ^S89A^ and USP14^C114A^ plasmids were constructed by site-directed mutagenesis using the inverse PCR from the TAZ-Flag and USP14-HA plasmids. The sequences of gene-specific small hairpin RNAs were designed and synthesized by Sangon Biotech and inserted into the pLKO.1 vector. The primer sequence information was listed in Table S7. Transient transfection was carried out by using polyethyleneimine (25 kDa, Polyscience, US) according to the manufacturer’s protocol. For the generation of stably transduced cell lines, HEK293T cells were cotransfected with the target gene plasmid, the pMD2.G envelope plasmid and the psPAX packaging plasmid. After 72 h, the supernatant was collected and filtered through a 0.45 µm filter (Biofly, Wuhan, China). SW1990 and PANC-1 cells were transduced by treatment with 1 ml of the viral supernatant in the presence of 1 μl of polybrene (40804ES76, Yeasen, Shanghai, China) for 8 h. Transduced cells were successively screened with puromycin dihydrochloride (1 μg/ml) (ST551, Beyotime, China) for 2 weeks. Next, immunoblotting and RT-qPCR were performed to determine the knockdown or overexpression efficiency of the indicated gene.

### Cell proliferation assay

Cell viability was examined with a Cell Counting Kit (CCK-8) (40203ES92, Yeasen, Shanghai, China). Briefly, stably transduced cells were plated into 96-well plates. CCK-8 reagent was added to the wells according to the manufacturer’s instructions. Cells were cultured for 1.5 h at 37 °C in 5% CO_2_, and the absorbance was measured at 450 nm with a Thermo Scientific™ Multiskan™ FC instrument (Thermo Fisher, Shanghai, China). For flow cytometry, CFSE [5(6)-carboxyfluorescein diacetate succinimidyl ester], an intracellular fluorescent dye that can be used to monitor cell division, was used. Briefly, cells were labelled with CFSE. After 48 h, cells were collected and analysed in a FACScan flow cytometer (BD Biosciences, US).

### Cell cycle assay

Cell cycle and apoptosis analysis kit (Yeasen, China) were used to detect the effect of USP14 on the cell cycle. PC cells were treated with the indicated treatment, harvested cell with trypsin-EDTA, centrifuged, resuspended 70% ethanol at −20 °C for 12 h, centrifuged, resuspended in a staining solution containing 5 μl PI solution and 5 μl RNase A solution, incubated in the dark at 37 °C for 30 min and analysed by flow cytometry.

### Cell apoptosis assay

PC cells were transfected with the indicated plasmid/treatment, and an Annexin V-EGFP/PI Apoptosis Detection Kit (Yeasen, China) was used to detect cell apoptosis according to the manufacturer’s protocol. Cells were harvested with trypsin free-EDTA, and resuspended in 1× Binding Buffer containing 2.5 μl of Annexin V-EGFP and 5 μl of PI Staining Solution. The cells were incubated at room temperature in the dark for 5 min and analysed by flow cytometry.

### Nuclear and cytoplasmic protein purification and isolation

Nuclear and cytoplasmic extraction reagents were used to separate the nuclear and cytoplasmic proteins according to the manufacturer’s protocol (78833, Thermo Fisher, China). Briefly, PC cells (2 × 10^6^) were transfected with the indicated plasmid/treatment, harvested with trypsin-EDTA, washed with PBS, centrifuged at 500 g for 5 min at 4 °C and the supernatant was discarded. Ice-cold CER I was added to the cell pellet, vortexed vigorously for 15 s and incubated for 10 min on ice. Then, ice-cold CER II was added to the tube, vortexed for 5 s and incubated on ice for 1 min, and vortexed for another 5 s. The tube was centrifuged for 5 min at maximum speed (16,000 g) at 4 °C in a microcentrifuge, and the supernatant containing the cytoplasmic extract was obtained. Next, ice-cold NER was added to the insoluble fraction, vortexed for 20 s every 10 min, for 40 min. The liquid was centrifuged at 16,000 g for 10 min at 4 °C, and the supernatant containing nuclear extract was obtained.

### Transwell assay and wound healing assay

For the transwell migration assay, cells containing 200 μl of serum-free DMEM were plated in the upper chambers of a transwell plate (Corning, US), and 700 μl of DMEM with 10% FBS was added to the lower chambers. After 32 h of coculture, the membranes were fixed with 4% paraformaldehyde and stained with crystal violet (C0121, Beyotime, China). The cells on the upper surface of the membrane were removed, and images were acquired under a microscope (Mshot, Guangzhou, China). The number of cells in 3 visual fields was counted, and the assay was repeated in triplicate. For the invasion assay, the transwell chamber membranes were coated with Matrigel (354234, BD Bioscience, US), and the remaining steps were the same as those for the Transwell migration assay.

For the wound healing assay, cells were seeded at 90% confluence, and a scratch was made in a straight line in the cell monolayer; the cells were then washed with PBS and cultured in FBS-free DMEM. After 72 h, the gap was photographed under a microscope.

### Immunofluorescence staining

SW1990 and PANC-1 cells were seeded on glass coverslips in 6-well plates and cotransfected with the TAZ-Flag and USP14-HA plasmids. After 48 h, the cells were fixed with 4% paraformaldehyde for 15 min, permeabilized with 0.1% Triton X-100 for 10 min, incubated with the primary antibody at 4 °C overnight, and incubated with the secondary antibody for 1 h at room temperature. The cells were visualized and photographed under a fluorescence microscope.

### Construction of USP14-knockout cell lines by CRISPR/Cas9 gene editing

The SW1990 USP14 knockout cell line was constructed with the CRISPR/Cas9 system. Briefly, the sgRNA (5’-TGAACCTCCAATGGTATTCA-3’) targeting the genomic sequence of USP14 exon 2 was designed using the Benchling website (https://benchling.com/) and inserted into the BbsI site in the PX459 vector (48139, Addgene, Watertown, US). SW1990 cells were transfected with the sgRNA construct USP14-sgRNA. At 48 h post-transfection, cells were selected with 1 μg/ml puromycin dihydrochloride for 3 days. Monoclonal cell lines were isolated using a limiting dilution method and further screened by Sanger sequencing (Tsingke, Wuhan, China). The correct clones were finally confirmed by immunoblotting analysis with a USP14-specific antibody.

### GSEA

The datasets (GSE15471, GSE28735, and GSE16515) were downloaded from GEO website (https://www.ncbi.nlm.nih.gov/geo/). The dataset of Hippo signalling pathway was downloaded from KEGG website (https://www.kegg.jp/). Samples were divided into a PDAC group and a paracarcinoma group according to the dataset information. The mRNA expressions of all genes were analysed with GSEA_4.0.3 software.

### GST pull-down assay

Full-length of TAZ/USP14 was generated by PCR from TAZ-Flag/USP14-HA plasmids and cloned into the pGEX-4T-1 vector. The GST/GST-TAZ/GST-USP14 plasmids were transfected into *Escherichia coli* BL21 and IPTG (100 μM final concentration) was added at 16 °C for 12 h. Then, RIPA lysis buffer (50 mM Tris pH 7.4, 150 mM NaCl, 0.5% NP40, 1 mM EDTA, protease inhibitor cocktail) was added to *E. coli*. The *E. coli* were ultrasonicated and centrifuge, and the supernatant was collected and incubated with glutathione agarose at 4 °C for 1 h on a rotating device. The glutathione agarose was incubated with cell lysis at 4 °C for 4 h on a rotating device. The bound proteins were eluted with RIPA lysis buffer and analysed by western blotting.

### ChIP-qPCR

ChIP assay was performed according to the instructions of the SimpleChIP® Enzymatic Chromatin IP Kit (Magnetic Beads) (#9003, Cell Signaling Technology, US). Briefly, cells are transfected with vector/TAZ-S89A plasmids, 1% formaldehyde (final concentration) was added to the medium, and proteins were cross-linked to DNA. Then glycine solution (#7005, Cell Signaling Technology, US) was added to the medium and the cells were collected. Lysis buffer was added to the cells to continue nuclear preparation and chromatin digestion. RNase A and Proteinase K were used to digest samples at 65 °C for 2 h. Purified DNA from samples, was incubated with the antibody overnight at 4 °C and incubated with Protein G Magnetic Beads for 2 h at 4 °C with rotation. The pelleted Protein G magnetic beads were washed, chromatin was eluted from Antibody/Protein G Magnetic Beads and crosslinking was reversed. DNA was purified by using spin columns, and quantitative PCR was used to analyse the results. The primer sequence information is listed in Table S8.

### Experiments with animals

The experiments with animals were conducted in accordance with the Association for Assessment and Accreditation of Laboratory Animal Care guidelines and approved by the Committee on Ethics of Animal Experiments of Huazhong University of Science and Technology. Four-week-old female BALB/c nude mice were purchased from Gempharmatech Co., Ltd (Jiangsu, China), and randomized (simple randomization) into groups (10 mice per group). Stably transduced cells were resuspended in 100 μl of PBS and injected subcutaneously into the right axillae (2 × 10^6^ cells per mouse) or the spleens of the nude mice (2 × 10^5^ cells per mouse), and semi-spleen sectioning was performed to generate the liver metastasis model. In brief, the spleens of BALB/c nude mice were cut into two halves, and cells were injected into one hemi-spleen which was removed 10 min after the injection to establish a liver metastasis model. Mouse body weight and tumour volume were measured every three days. The mice were treated with IU1 (MedChemExpress, HY-13817) (40 mg/kg in 150 μl of corn oil) or corn oil by intragastric administration every three days when the tumour volume reached 150 mm^3^ or one week after spleen injection. Some mice were sacrificed by cervical dislocation 21 days after the hemi-spleen injection. The number of metastatic nodules on the surface of the liver was counted with a diameter greater than 1.0 mm [48]. Tumours, livers and lungs were collected and fixed with 4% paraformaldehyde for histological analysis. Some mice continued to be fed, and the OS time was determined.

### TMA analysis

TAZ and USP14 expression in human PDAC TMA was scored in the nucleus and cytoplasm by two experienced pathologists. Three visual fields were randomly selected for each sample, and scored according to the percentage of positive cells and staining intensity under a microscope. The percentage of positive cells in all counted cells was divided into five groups (< 5% score zero points; 5%~25% score one point; 26%~50% score two points; 51%~75% score three points; > 75% score four points). Staining intensity was divided into four groups (zero points for negative; one point for weak; two points for moderate; three points for strong). Final score = score (percentage of positive cells in cytoplasm) × score (staining intensity in cytoplasm) + score (percentage of positive cells in nucleus) × score (staining intensity in nucleus).

### Statistical analysis

All western blot and flow cytometry analyses (CFSE assay, cell cycle and cell apoptosis assay) were performed with three independent experiments. All luciferase reporter assay, CCK8 assay, qRT-PCR, transwell assay, and wound healing assay were performed with at least three biological replicates and three independent experiments. Statistical analysis was performed using SPSS 26.0 (SPSS Inc, Chicago, IL, USA). All experiments were independently carried out at least three times, and data are presented as the mean ± SD unless otherwise indicated. Statistical analysis was performed with a two-tailed Student’s *t* test unless otherwise indicated, and *P* values < 0.05 were considered statistically significant. *P < 0.05; **P < 0.01; ***P < 0.001.

## Supplementary information

Supplementary Figure and Table Legend

Supplementary Figure 1

Supplementary Figure 2

Supplementary Figure 3

Supplementary Figure 4

Supplementary Figure 5

Supplementary Figure 6

Supplementary Figure 7

Supplementary Figure 8

Supplementary Figure 9

Supplementary Table

Author Contribution form

Reproducibility Checklist

Original western blots

## Data Availability

The datasets used and analysed during the current study are available from the corresponding author on reasonable request.
