# Supplementary material for: A self-amplifying USP14-TAZ loop drives the progression and liver metastasis of pancreatic ductal adenocarcinoma
Source: Cell Death Differ. 2022 Jul 29;30(1):1–15. doi: 10.1038/s41418-022-01040-w (PMC9883464; doi:10.1038/s41418-022-01040-w)
Supplement: Supplementary file 1 — Supplementary Figure and Table Legend [file 41418_2022_1040_MOESM1_ESM.docx]

**Supplementary Figure and Table Legends**

**Supplementary Figure 1. YAP and TAZ were overexpressed in pancreatic cancer.**

A-B. GSEA demonstrated that Hippo pathway signalling was significantly changed in PDAC tissue compared with paracarcinoma tissue in the GEO datasets (GSE28735 and GSE16515).

C-H. The expression of YAP/TAZ was significantly upregulated in PDAC tumour tissues compared with both paired (GSE15471 and GSE28735) and unpaired (GSE16515) paracarcinoma issues. The data were analysed by paired two-tailed Student’s *t* test for paired cohorts and two-tailed Student’s *t* test for the unpaired cohort.

I. Individuals in the TCGA-PAAD dataset were divided into the YAP^high^ expression and YAP^low^ expression groups according to the median expression level of YAP. The overall survival rate of the PAAD patients was estimated by Kaplan-Meier analysis.

**Supplementary Figure 2. USP14 was negatively correlated with Hippo signalling in pancreatic cancer.**

A-B. Immunohistochemistry staining of TAZ and USP14 were shown in human PDAC tissue microarrays.

C. PANC-1 cells were transfected with plasmids encoding 81 DUBs, and immunoblotting analysis was performed to examine the expression level of TAZ.

D-G. The correlation between the mRNA expression of USP14 and that of TAZ target genes (*CYR61*, *CTGF*, *ANKRD1* and *BIRC5*) was analysed in the TCGA-PAAD dataset via Spearman correlation analysis.

**Supplementary Figure 3. USP14 regulated TAZ and TAZ target genes.**

A-B. Immunoblotting and RT–qPCR were used to measure the protein and mRNA expression levels of TAZ and TAZ target genes in SW1990 cells transfected with the USP14-HA (vector was used as a negative control) or shRNA against USP14 plasmids (shscramble was used as a negative control).

C. Schematic diagram of USP14 knockout using CRISPR/Cas9 technology (top panel) and the sequencing results of USP14 knockout cell clones (bottom panel) were showed.

D. Immunoblotting analysis of the core components of the Hippo signalling pathway were performed in SW1990 cells transfected with the USP14-HA or shRNA against USP14 plasmids.

**Supplementary Figure 4. USP14 interacted with TAZ.**

A-B. HEK293T cells were transfected with an empty vector, the USP14-HA plasmid or the TAZ-Flag plasmid. After 48 h, the cell lysates were collected, and a Co-IP assay was performed with the indicated antibody. The interaction was analysed by immunoblotting.

C-D. SW1990 cells were transfected with USP14-HA or TAZ-Flag plasmids. After 48 h, cell lysates were collected, and a co-immunoprecipitation (Co-IP) assay was performed with the indicated antibody. The interaction of USP14 and TAZ was analysed by immunoblotting.

E. PANC-1 cells were cotransfected with the USP14-HA and TAZ-Flag plasmids. After 48 h transfection, an immunofluorescence assay was conducted, and images were acquired by fluorescence microscopy.

F. PANC-1 cells were separated into the nucleus and cytoplasm by a nuclear and cytoplasmic protein extraction kit, Co-IP experiments were conducted in the cytoplasm or nucleus, and the interaction of TAZ and USP14 was detected by the Co-IP assay.

G-H. SW1990 cells were transfected with the USP14^C114A^-HA plasmid as indicated. Immunoblotting and RT–qPCR were performed to examine the protein (G) and mRNA (H) expression of TAZ and USP14.

**Supplementary Figure 5. USP14 promoted pancreatic tumour growth.**

A-B. PANC-1 and SW1990 cells with stable overexpression of USP14 were planted into 96-well plates (3000 cells per well). The cell viability was examined by CCK-8 kit and the absorbance was measured at 450 nm according to the indicated time.

C-D. PANC-1 and SW1990 cells with stable knockdown of USP14 were planted into 96-well plates (3000 cells per well). The cell viability was examined by CCK-8 kit and the absorbance was measured at 450 nm according to the indicated time.

E-F. PANC-1 and SW1990 cells with stable knockdown of USP14 were stained with the PI solution and RNase A solution, and flow cytometry was used to detect the change in the cell cycle. The experiment was independently repeated three times.

G-H. PANC-1 and SW1990 cells with stable knockdown of USP14 were stained with Annexin V-EGFP/PI solution, then the flow cytometry was used to detect the change in cell early and late apoptosis. The experiment was independently repeated three times.

I-J. Tumour volumes were measured at 2-day intervals refer to Fig 4B and 4H. The data were analysed by one-way ANOVA and were presented as the mean ± SD values.

**Supplementary Figure 6. USP14 promoted pancreatic tumour metastasis.**

A-B. PANC-1 and SW1990 cells with stable knockdown or overexpression of USP14 were constructed, and transwell assay was used to measure the migration and invasion ability of cells.

C-D. PANC-1 and SW1990 cells with stable knockdown or overexpression of USP14 were planted into 6-well plate at 90% confluence, and the wound healing assay was used to measure the migration of cells.

E-F. SW1990 cells with stable overexpression or knockdown of USP14 were injected into the spleens of nude mice. After 30 days, the lungs were excised. The H&E staining results of the lungs were shown (n = 10).

**Supplementary Figure 7. USP14 promoted pancreatic cancer progression in a TAZ-dependent manner *in vitro*.**

A. Immunoblotting was performed to examine the protein and mRNA expression of TAZ and USP14 in the PANC-1 and SW-1990 cells with stable USP14 overexpression and TAZ knockdown.

B: Immunoblotting and RT-qPCR were performed to examine the protein and mRNA expression of TAZ and USP14 in the PANC-1 and SW-1990 cells with stable TAZ knockdown.

C. CFSE staining and flow cytometry were performed to examine the proliferation of cells. The experiment was independently repeated three times.

D. Transwell assays were used to evaluate the migration and invasion abilities of the PANC-1 and SW1990 cells with USP14 overexpression and TAZ knockdown. The experiment was independently repeated three times.

**Supplementary Figure 8. USP14 was overexpressed in PDAC samples**

A-C. The expression of USP14 was analysed in PDAC tumour tissues compared with both paired (GSE15471 and GSE28735) and unpaired (GSE16515) paracarcinoma tissues in GEO datasets. The data were analysed by paired two-tailed Student’s *t* test for paired cohorts and two-tailed Student’s *t* test for the unpaired cohort.

D. Individuals in the TCGA-PAAD dataset were divided into the USP14^high^ expression and USP14^low^ expression groups according to the median expression level of USP14. The overall survival rate of the PAAD patients was estimated by Kaplan-Meier analysis.

E. Immunoblot analysis was used to examine the protein expression of USP14 in PDAC and paired adjacent tumour tissues.

F. RT-qPCR was used to measure the relative mRNA expression level of USP14 in PDAC and paired adjacent tumour tissues.

G. Immunoblotting analysis was used to examine the protein expression of USP14 in pancreatic cancer cell lines. Quantification of the USP14 protein level normalized to the GAPDH level was shown above the blot image. The experiment was independently repeated three times.

H. IHC staining of USP14 was performed on human PDAC TMAs (refer to Figure S2B), and representative images were presented.

I. The expression level of USP14 was calculated in PDAC and non-tumour tissues based on PDAC TMAs. The data were analysed by the two-tailed Student’s *t* test.

J. The expression level of USP14 and TAZ were counted in PDAC and non-tumour tissues based on PDAC TMAs. Pearson correlation analysis was performed to examine the correlation of USP14 expression and TAZ expression based on human PDAC TMAs (refer to Fig S2A-B).

**Supplementary Figure 9. YAP positively regulated USP14 expression at the transcriptional level**

A-B. SW1990 and PANC-1 cells were transfected with PLKO.1/YAP targeting knockdown plasmids, and immunoblotting and RT–qPCR were used to measure the protein and mRNA expression levels of USP14 and YAP.

C-D. SW1990 and PANC-1 cells were transfected with the vector/YAP-Flag plasmids, and immunoblotting and RT–qPCR were used to measure the protein and mRNA expression levels of USP14 and YAP.

**Supplementary Table 1.** Clinicopathological characteristics of patient samples and expression of USP14 in pancreatic cancer.

**Supplementary Table 2.** Correlation between USP14 expression and clinicopathologic characteristics of pancreatic cancer patients.

**Supplementary Table 3.** Univariate analysis of various prognostic parameters in patients with pancreatic cancer cox-regression analysis.

**Supplementary Table 4.** Multivariate analysis of various prognostic parameters in patients with pancreatic cancer cox-regression analysis.

**Supplementary Table 5.** Information for antibodies used in this study.

**Supplementary Table 6.** Primers for quantitative PCR.

**Supplementary Table 7.** Targeting sequences for shRNAs.

**Supplementary Table 8.** Primers for ChIP-qPCR.
