# Supplementary figures and images for: A self-amplifying USP14-TAZ loop drives the progression and liver metastasis of pancreatic ductal adenocarcinoma
Source: Cell Death Differ. 2022 Jul 29;30(1):1–15. doi: 10.1038/s41418-022-01040-w (PMC9883464; doi:10.1038/s41418-022-01040-w)

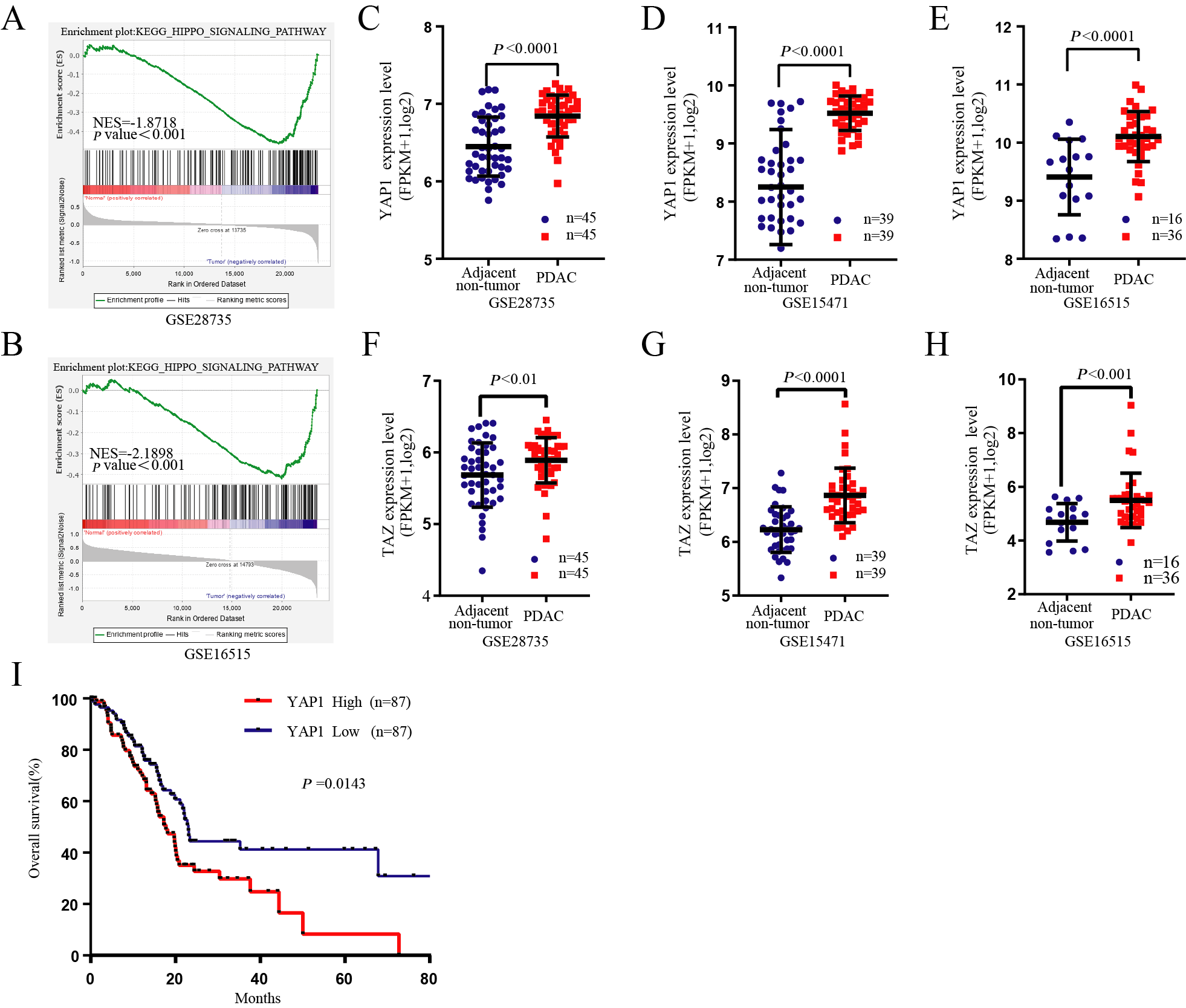

Supplement: Supplementary file 2 — Supplementary Figure 1 [file 41418_2022_1040_MOESM2_ESM.png]

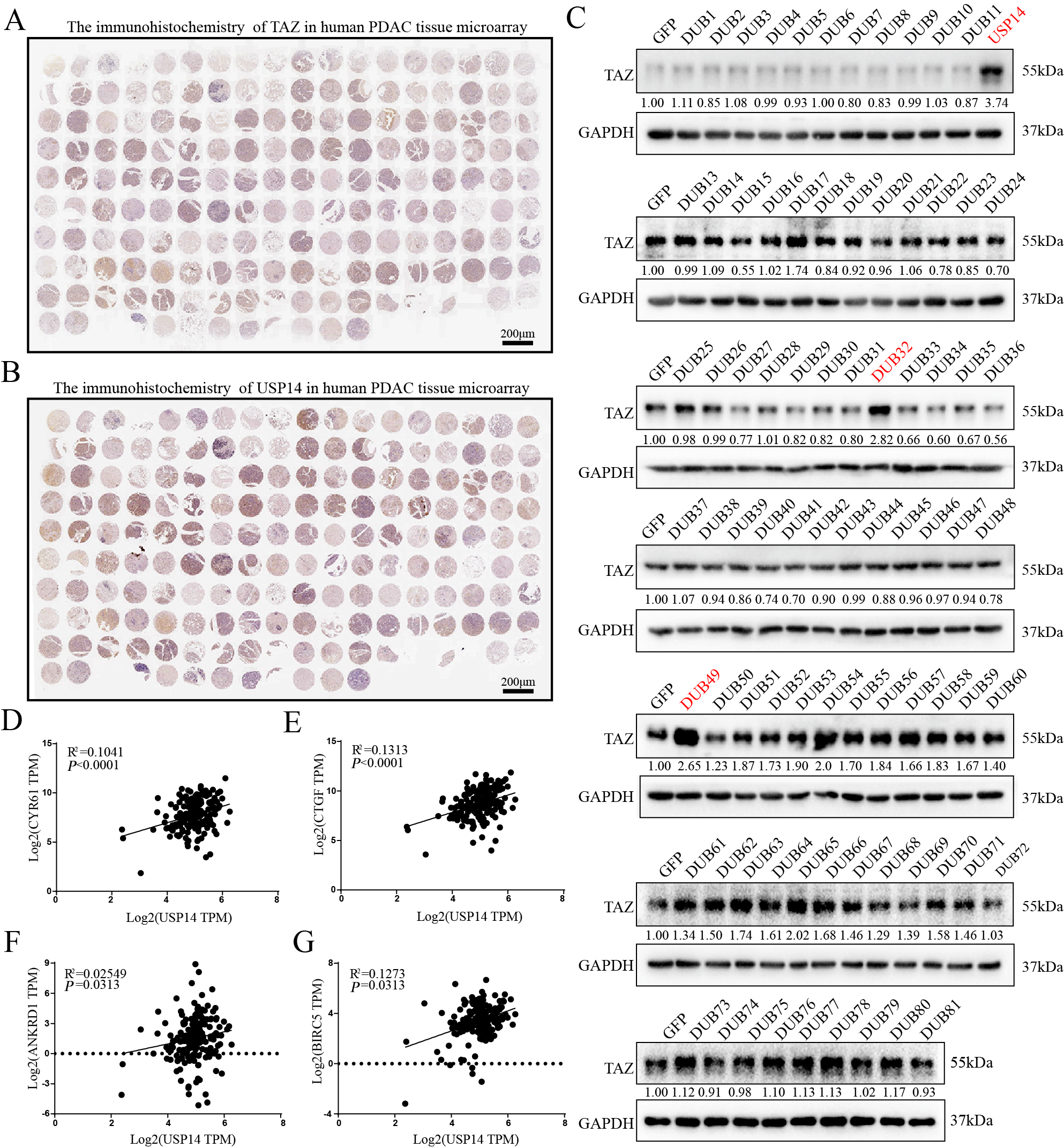

Supplement: Supplementary file 3 — Supplementary Figure 2 [file 41418_2022_1040_MOESM3_ESM.png]

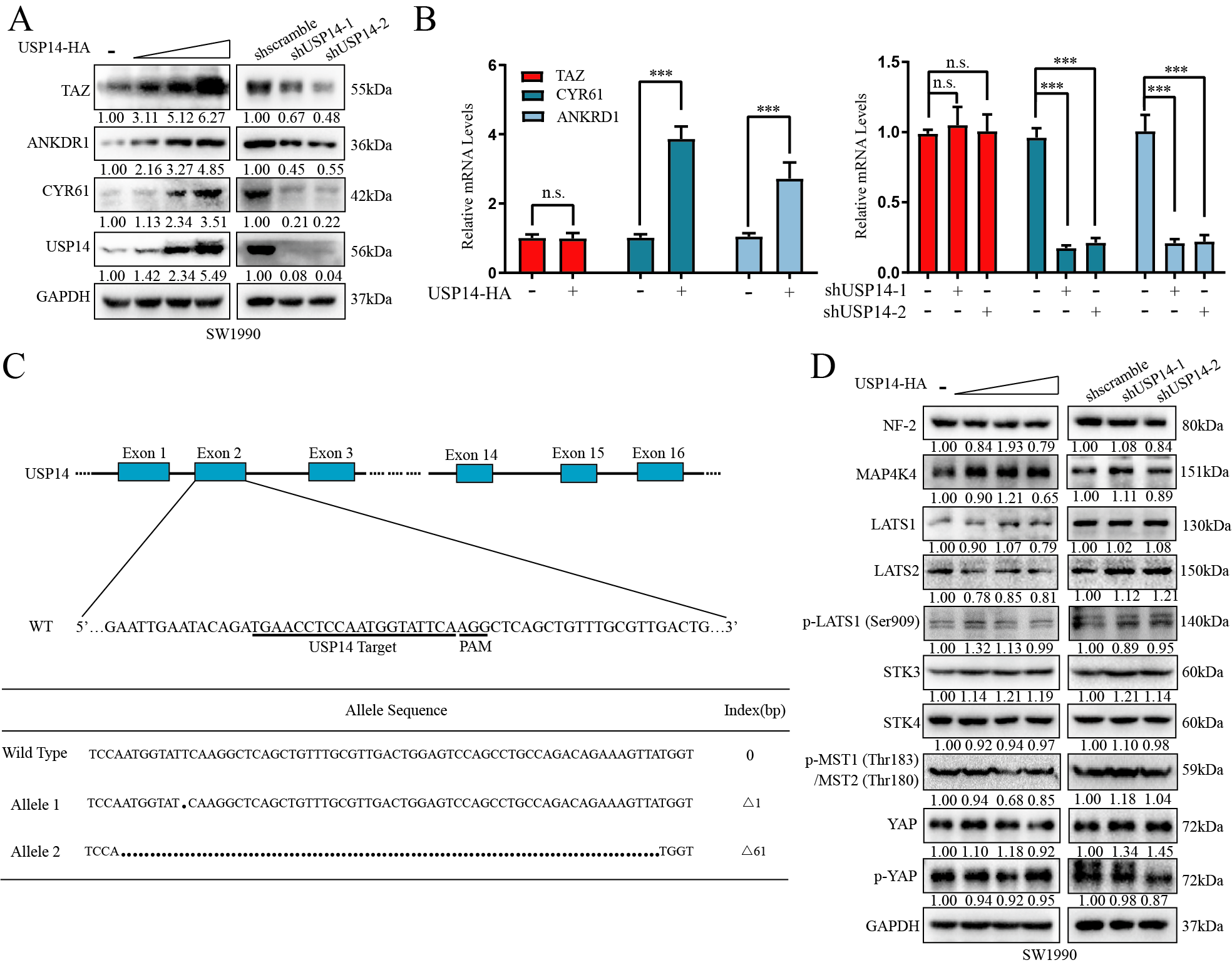

Supplement: Supplementary file 4 — Supplementary Figure 3 [file 41418_2022_1040_MOESM4_ESM.png]

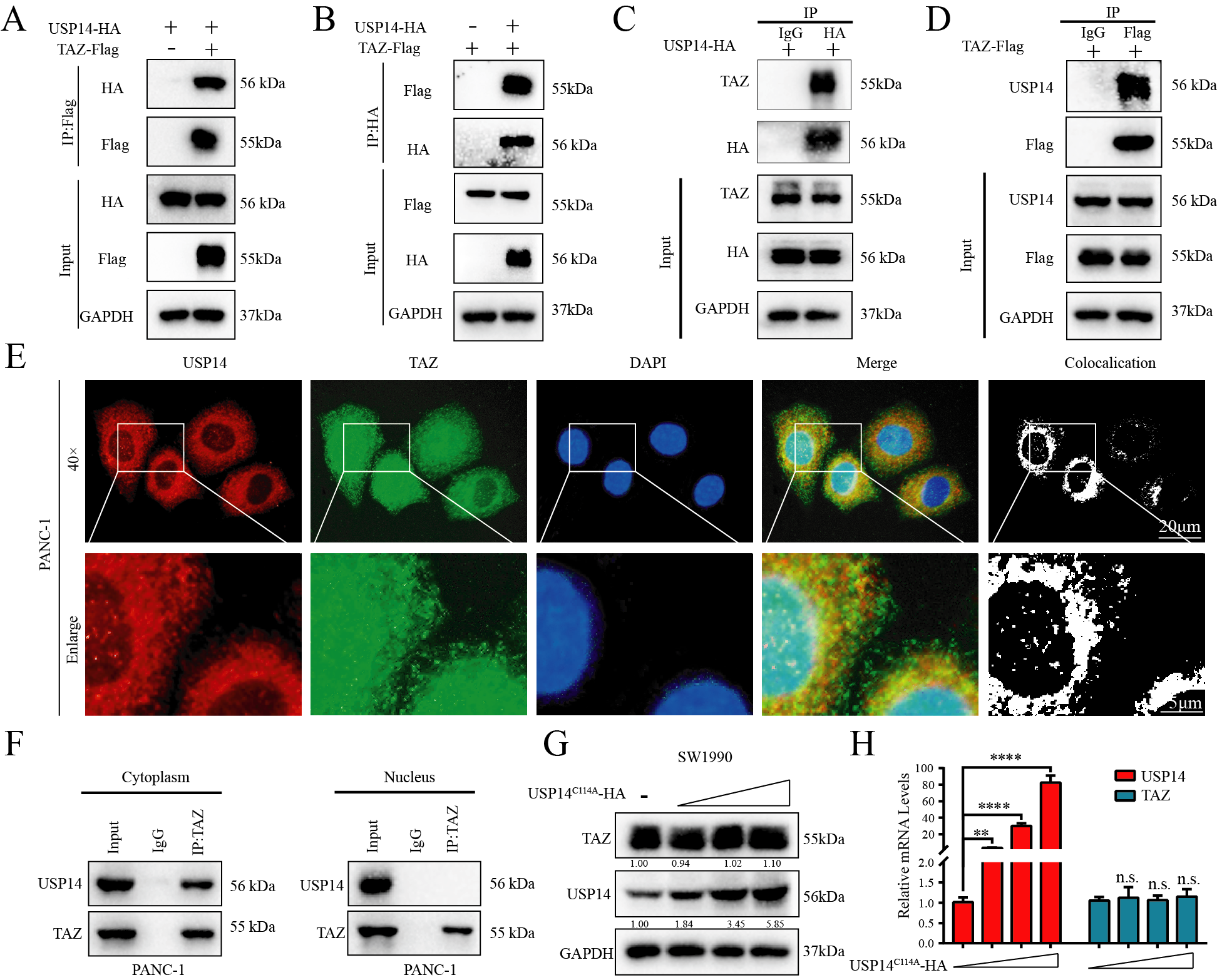

Supplement: Supplementary file 5 — Supplementary Figure 4 [file 41418_2022_1040_MOESM5_ESM.png]

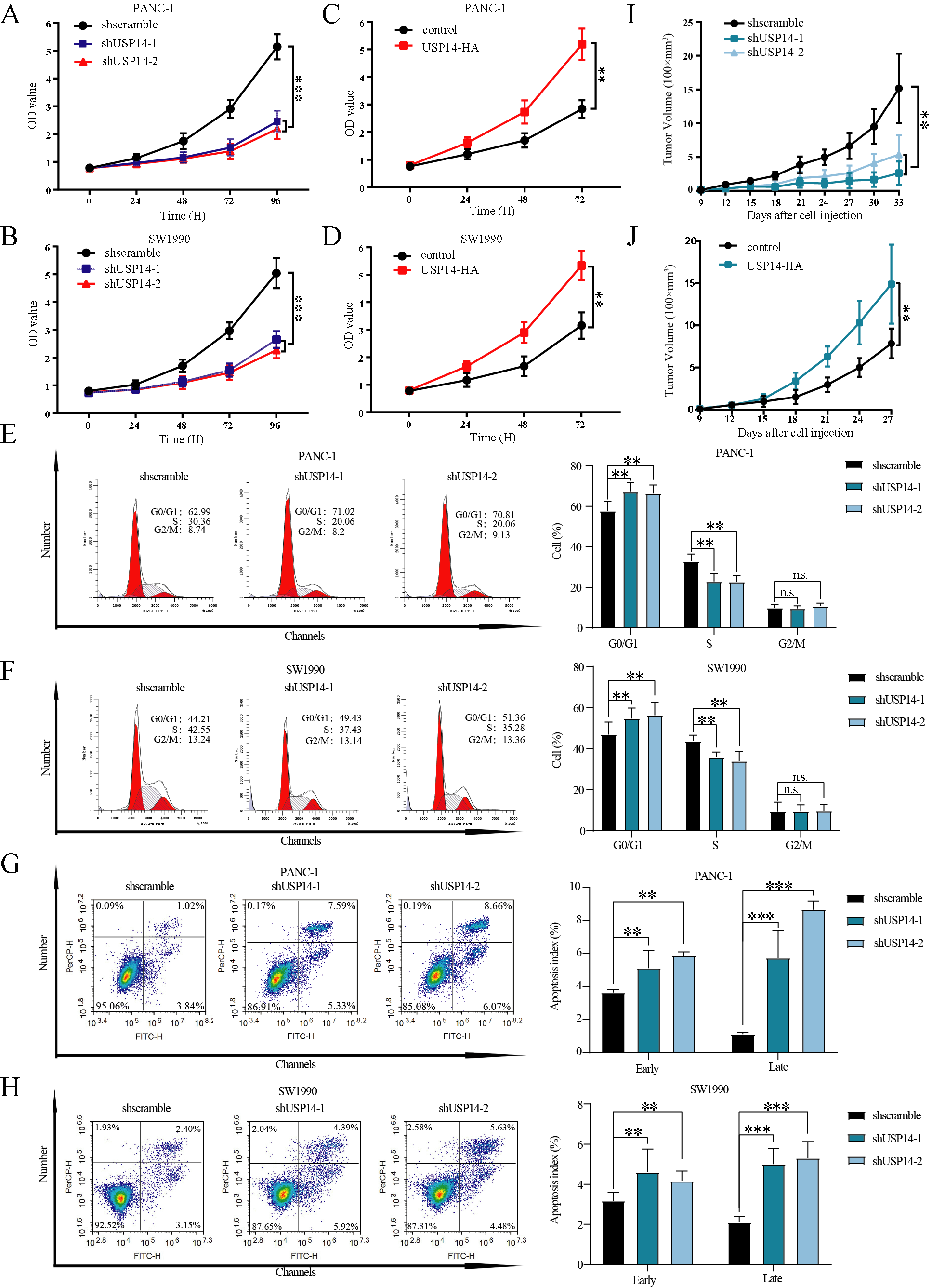

Supplement: Supplementary file 6 — Supplementary Figure 5 [file 41418_2022_1040_MOESM6_ESM.png]

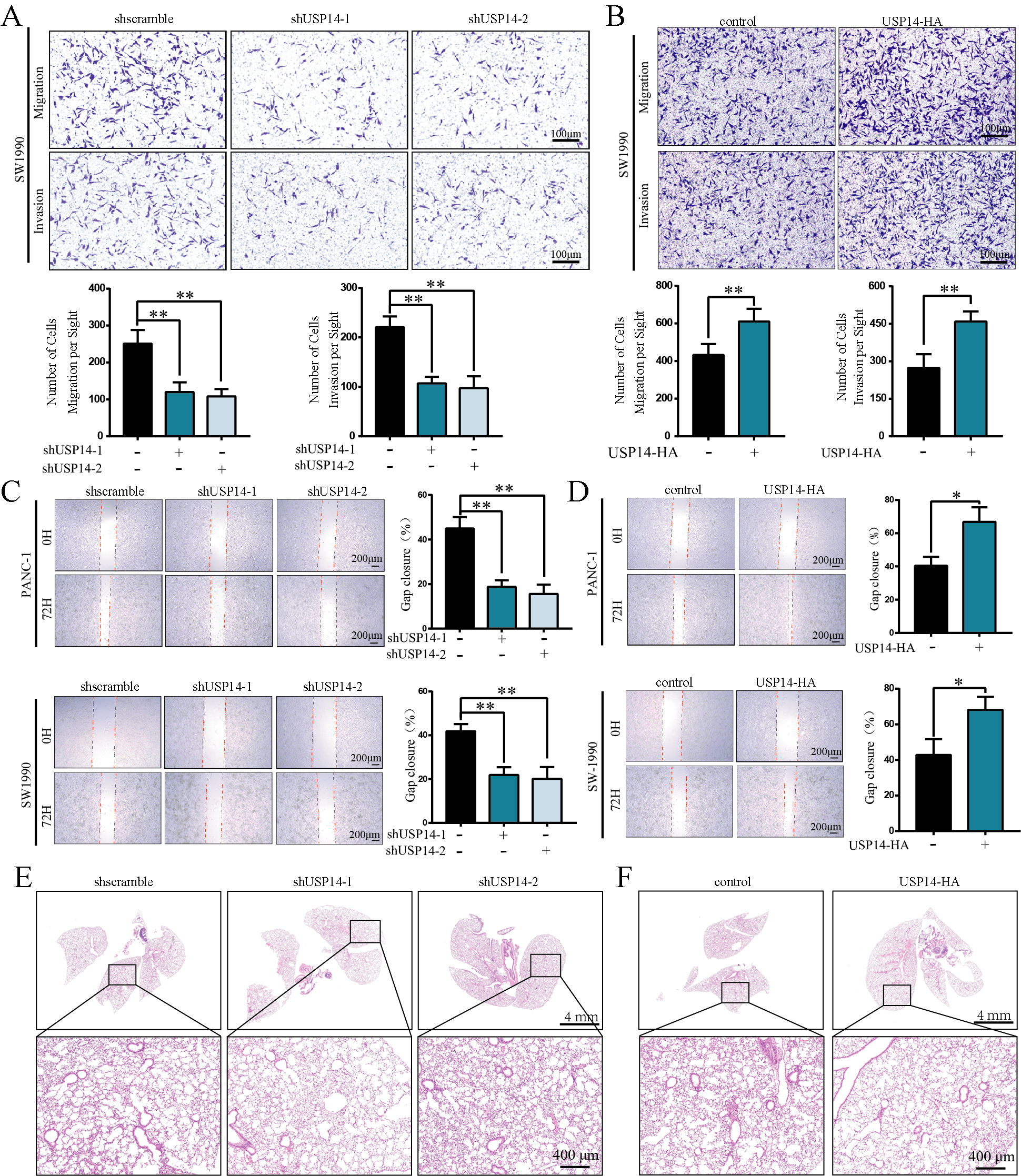

Supplement: Supplementary file 7 — Supplementary Figure 6 [file 41418_2022_1040_MOESM7_ESM.png]

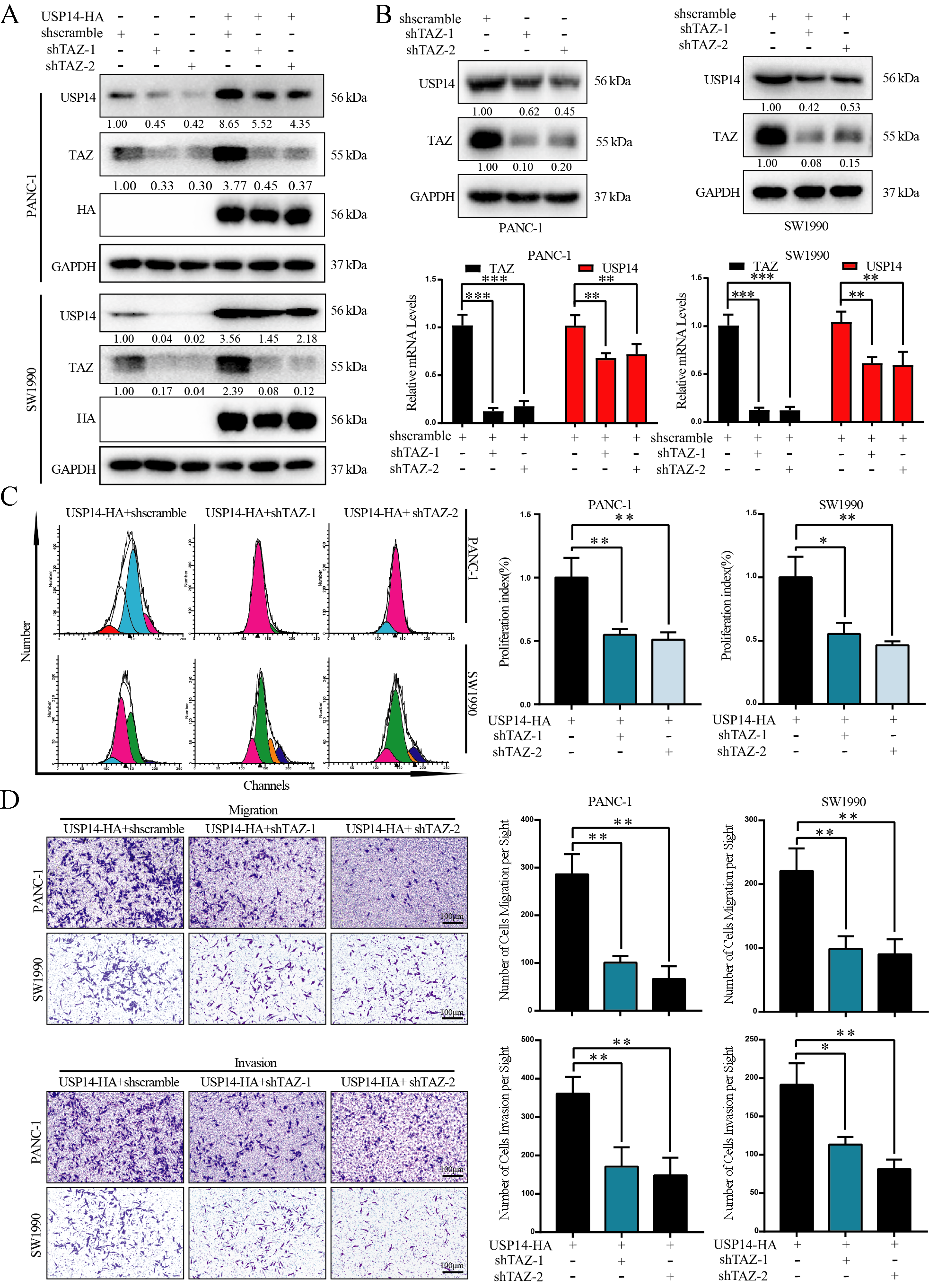

Supplement: Supplementary file 8 — Supplementary Figure 7 [file 41418_2022_1040_MOESM8_ESM.png]

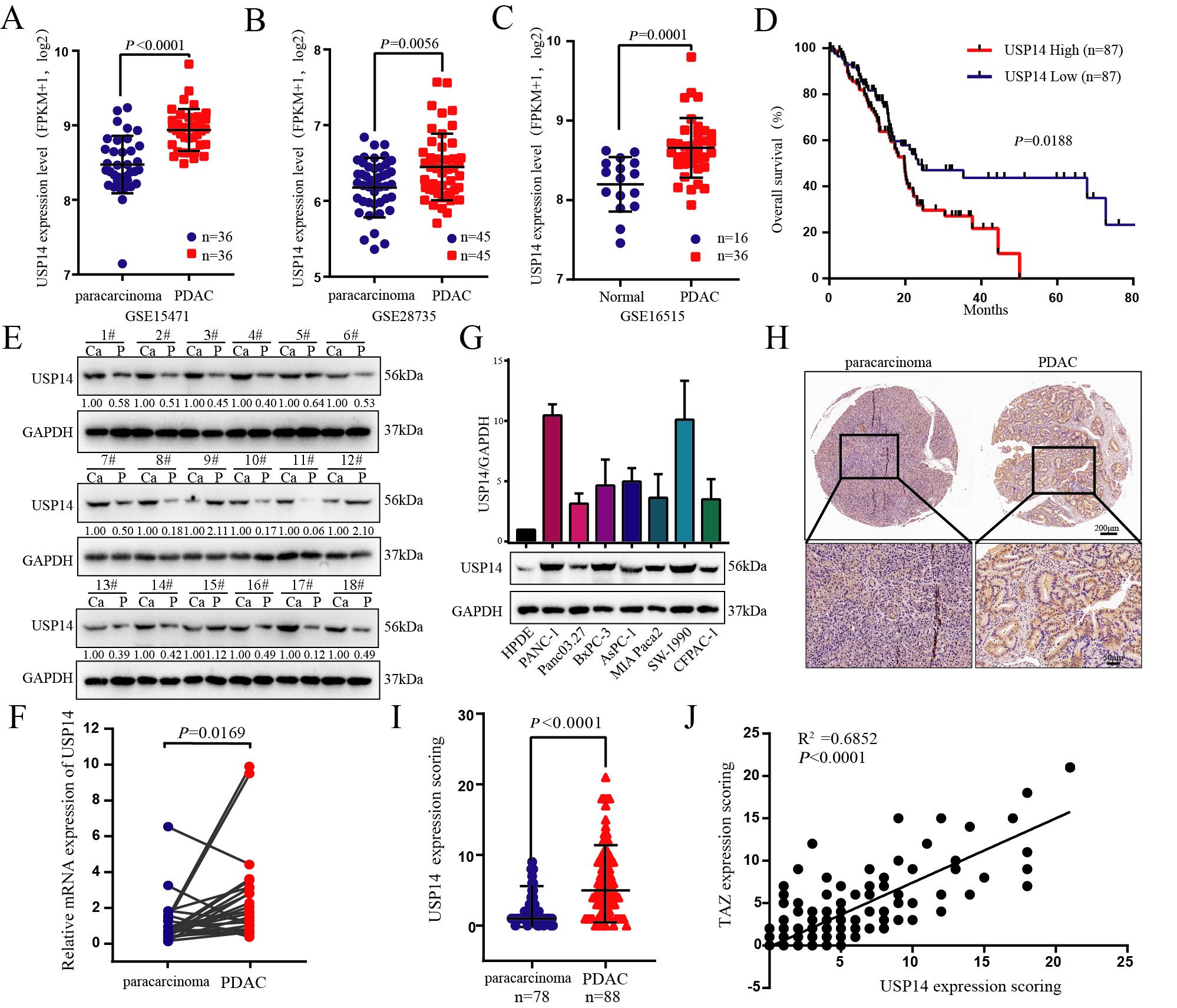

Supplement: Supplementary file 9 — Supplementary Figure 8 [file 41418_2022_1040_MOESM9_ESM.png]

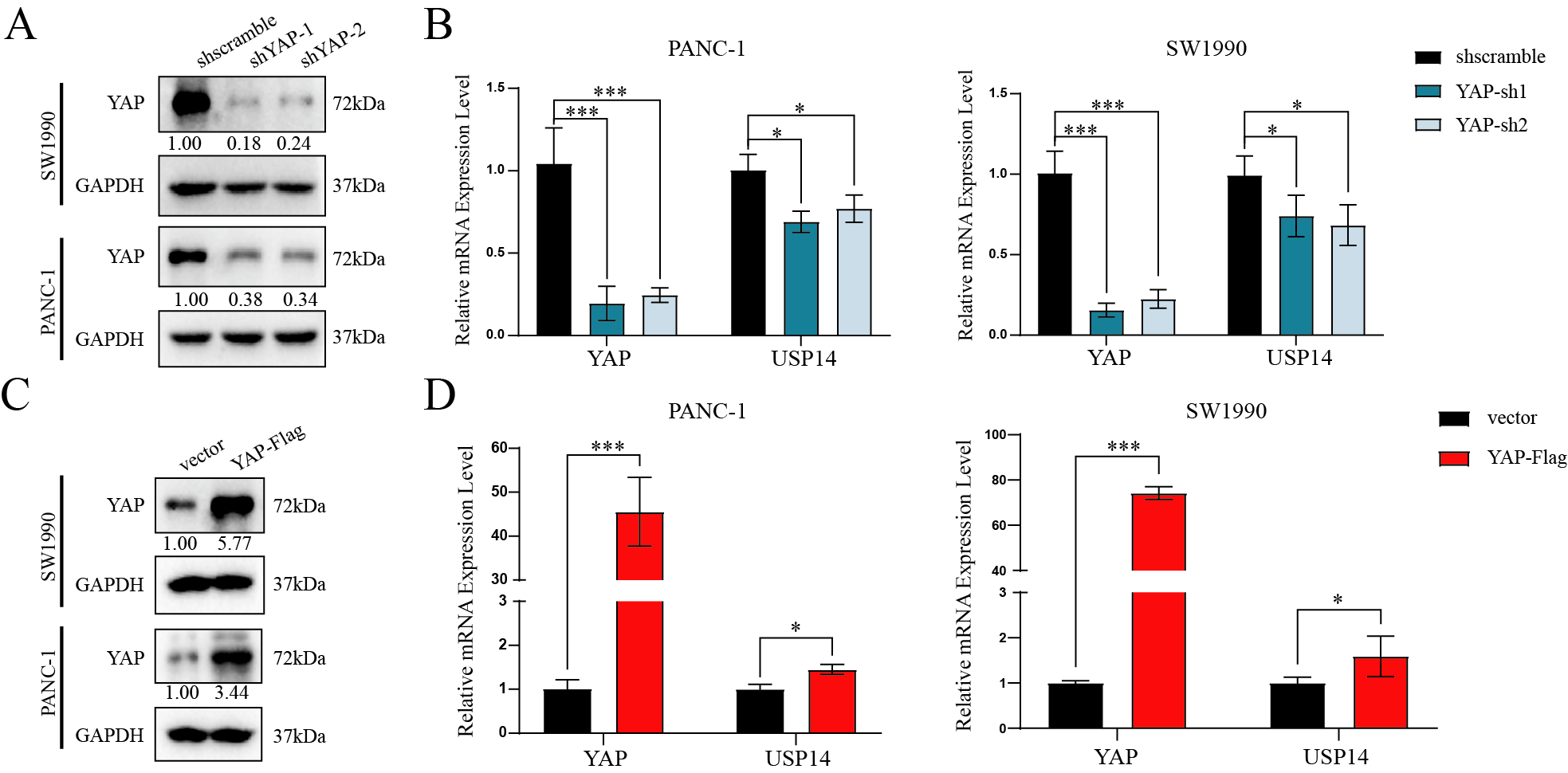

Supplement: Supplementary file 10 — Supplementary Figure 9 [file 41418_2022_1040_MOESM10_ESM.png]

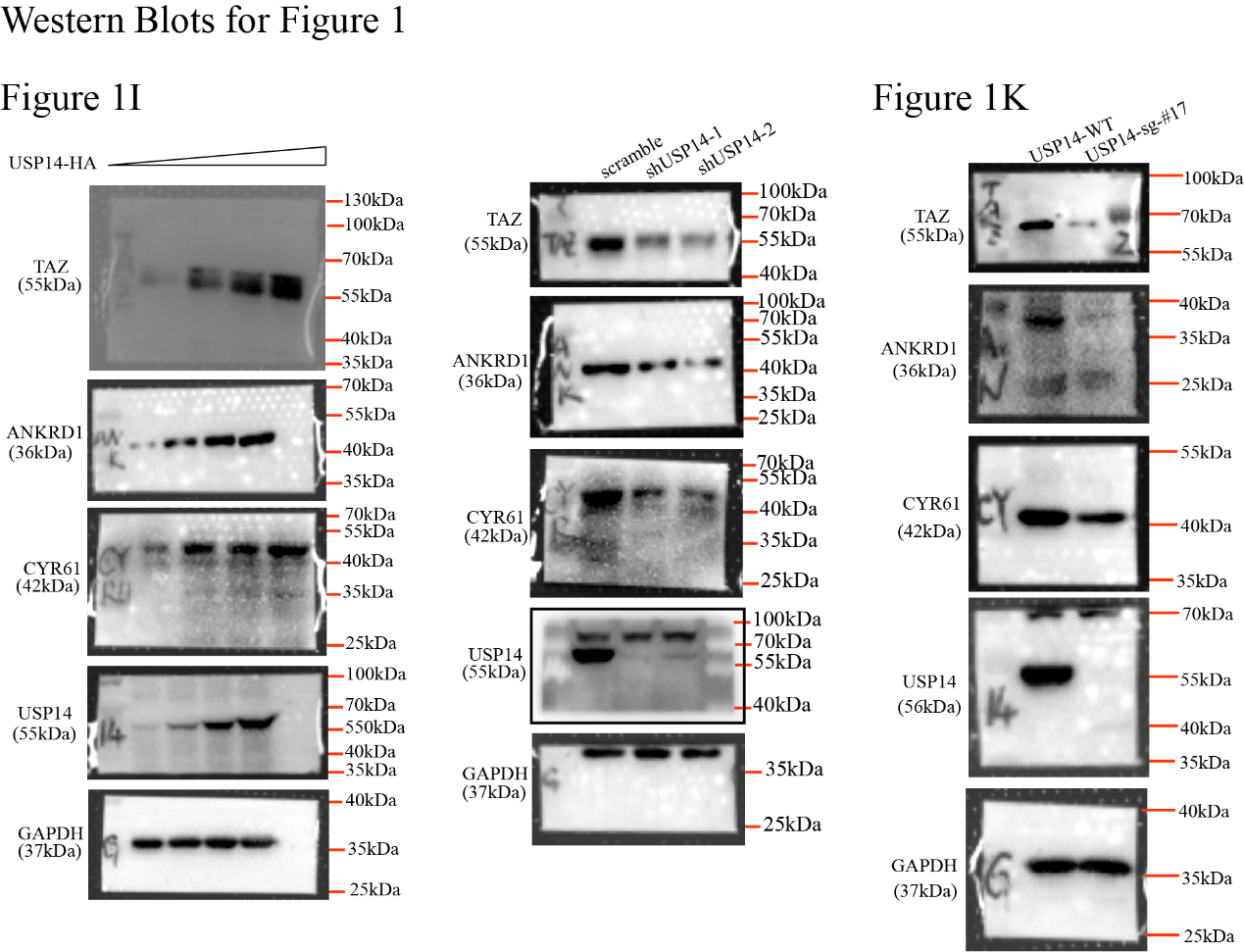


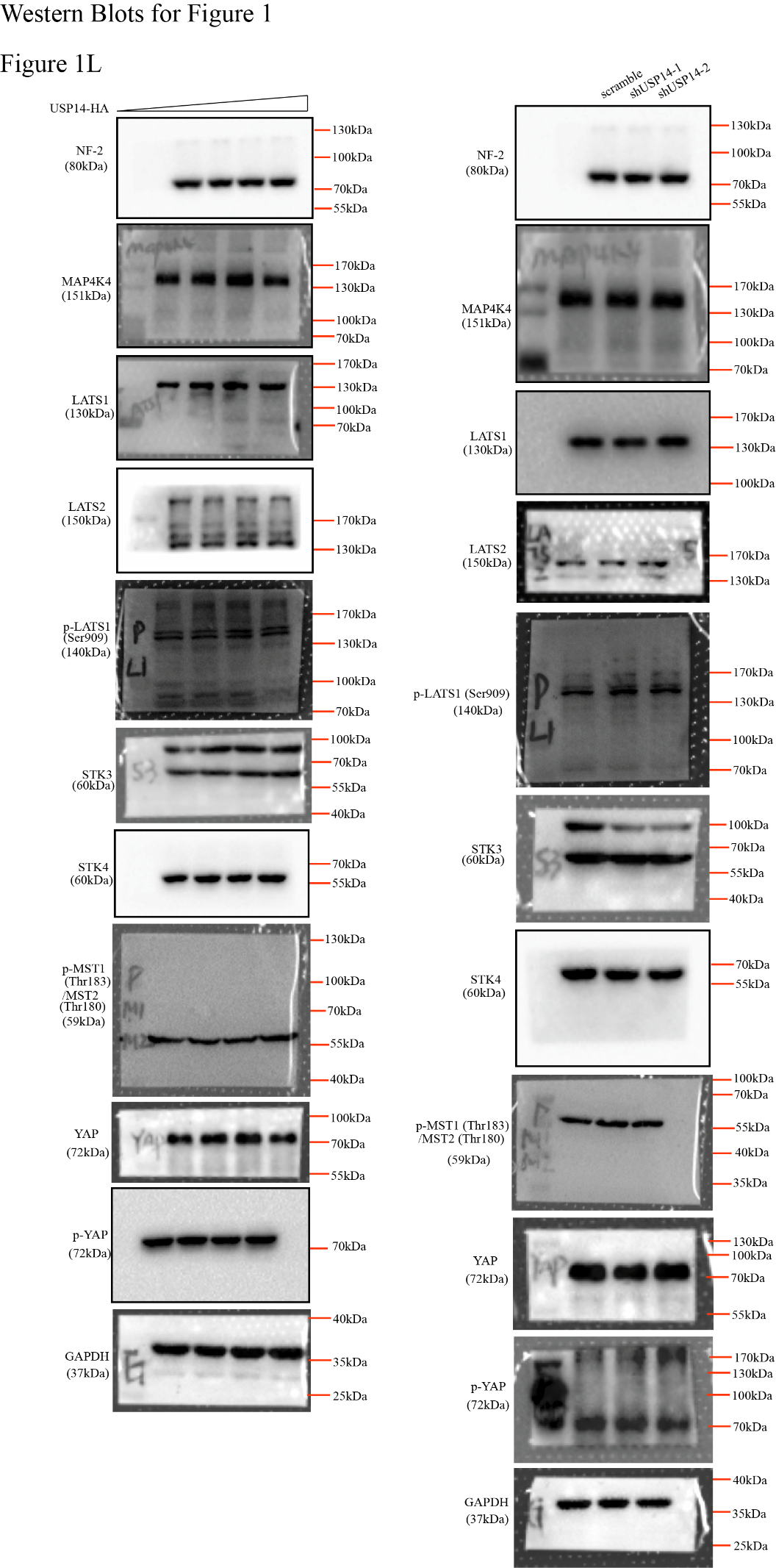


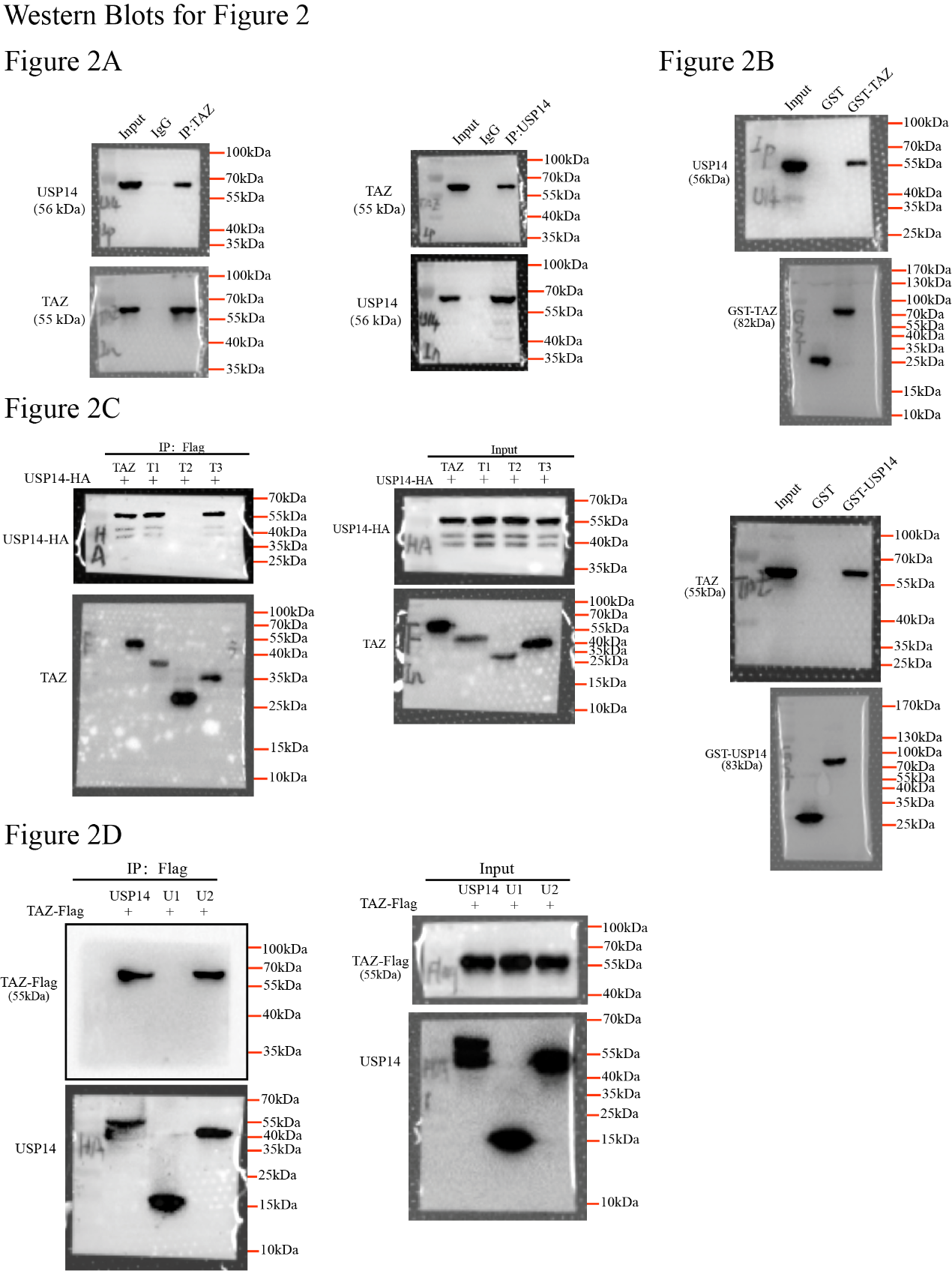


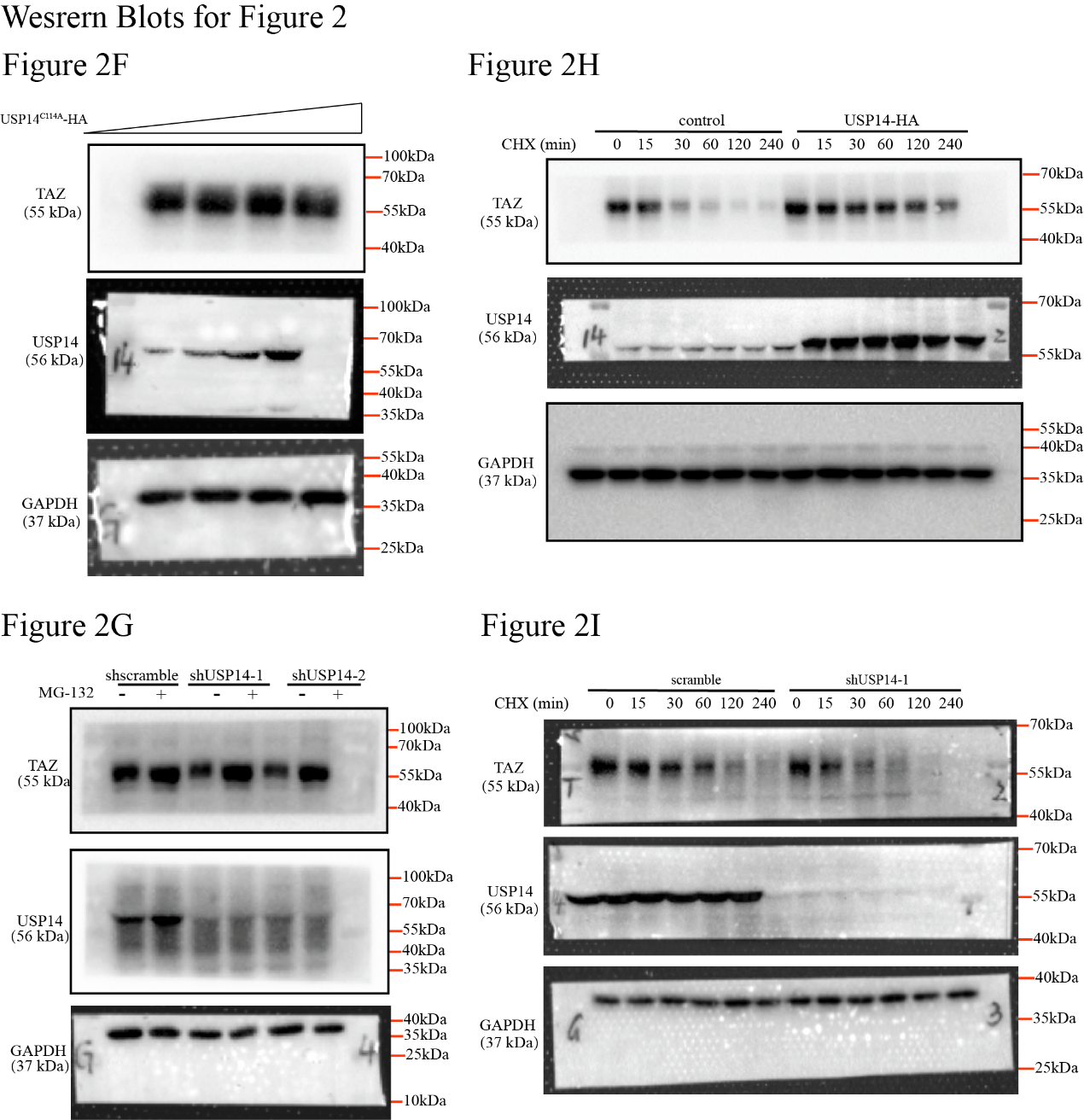


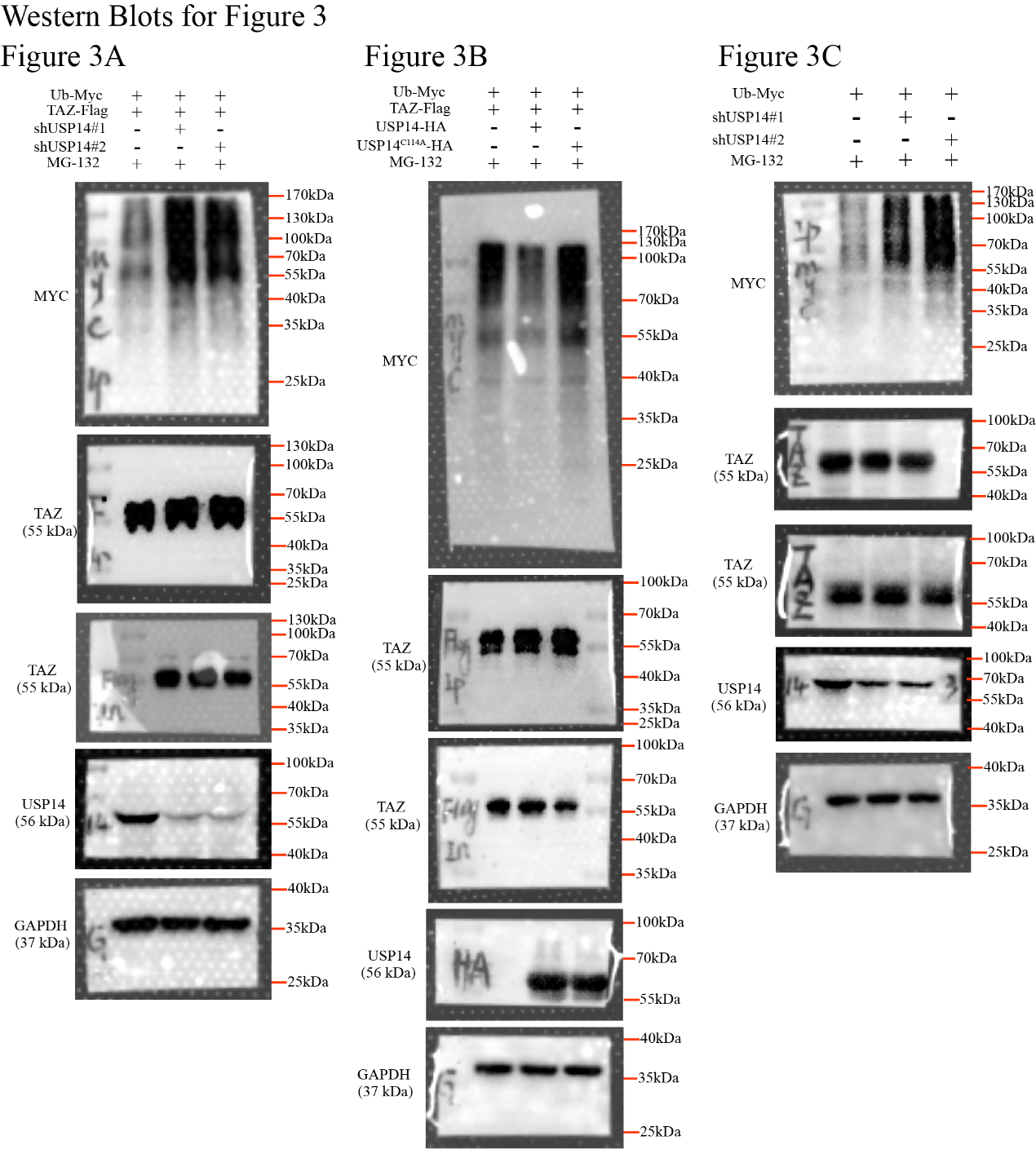

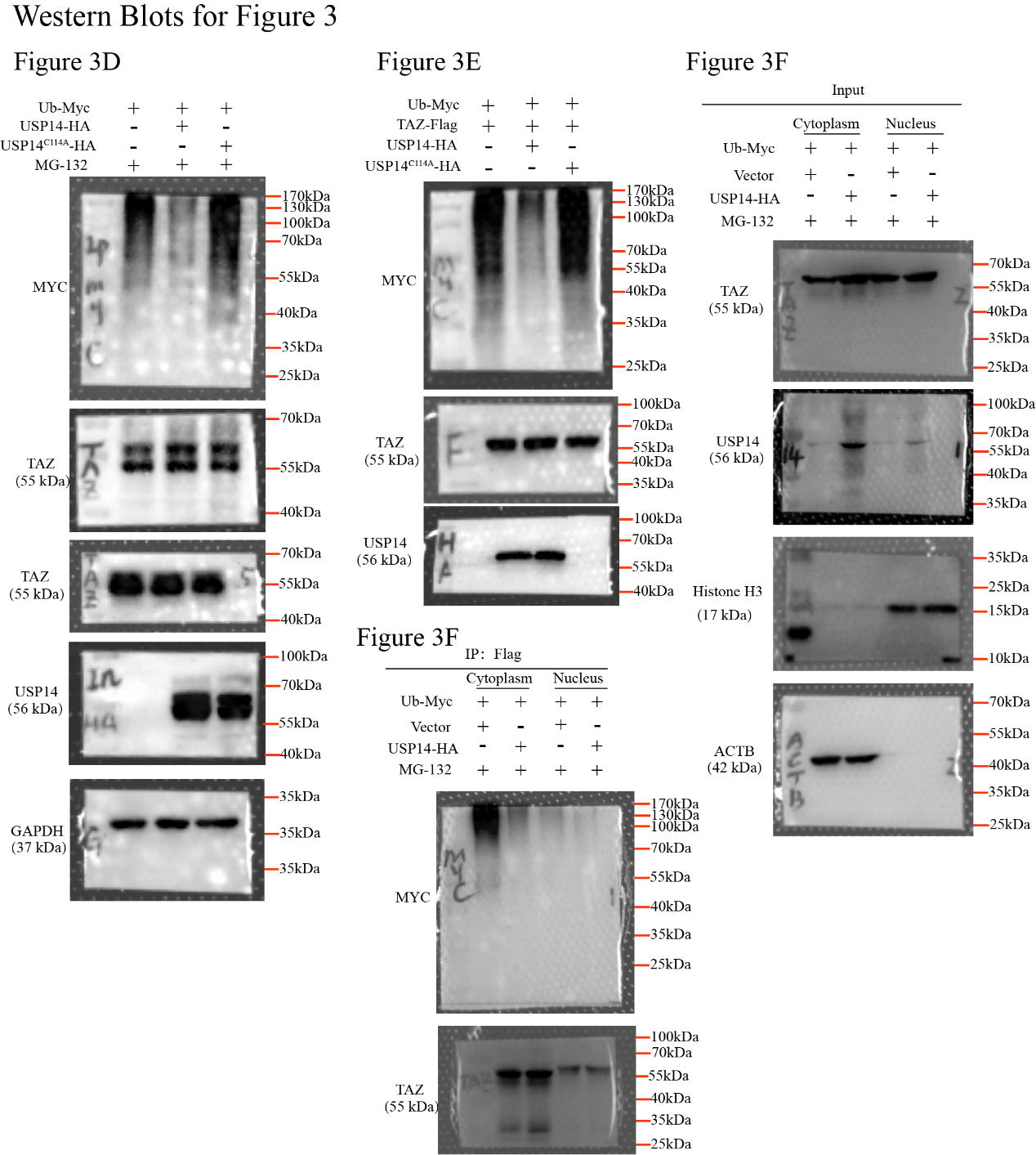

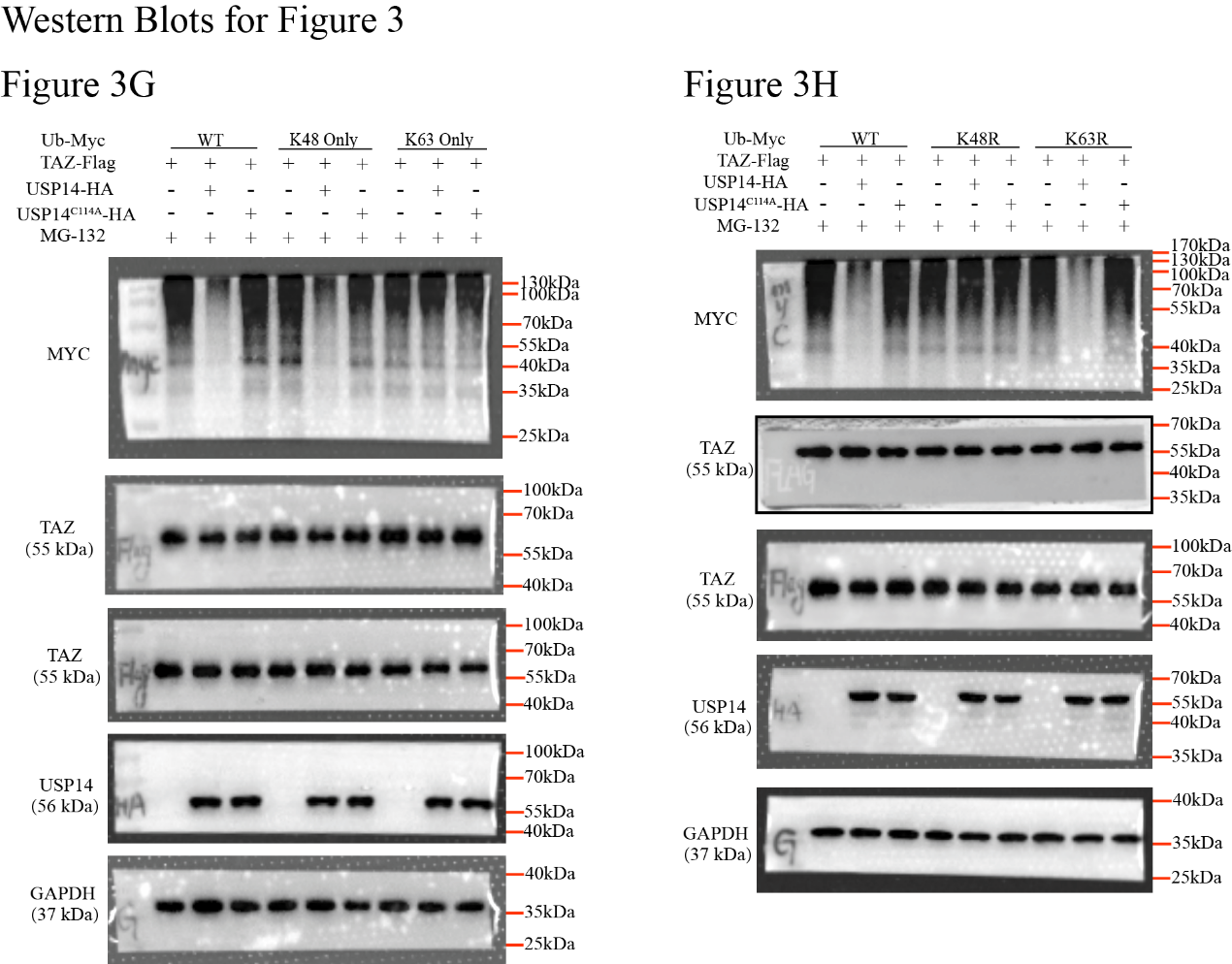


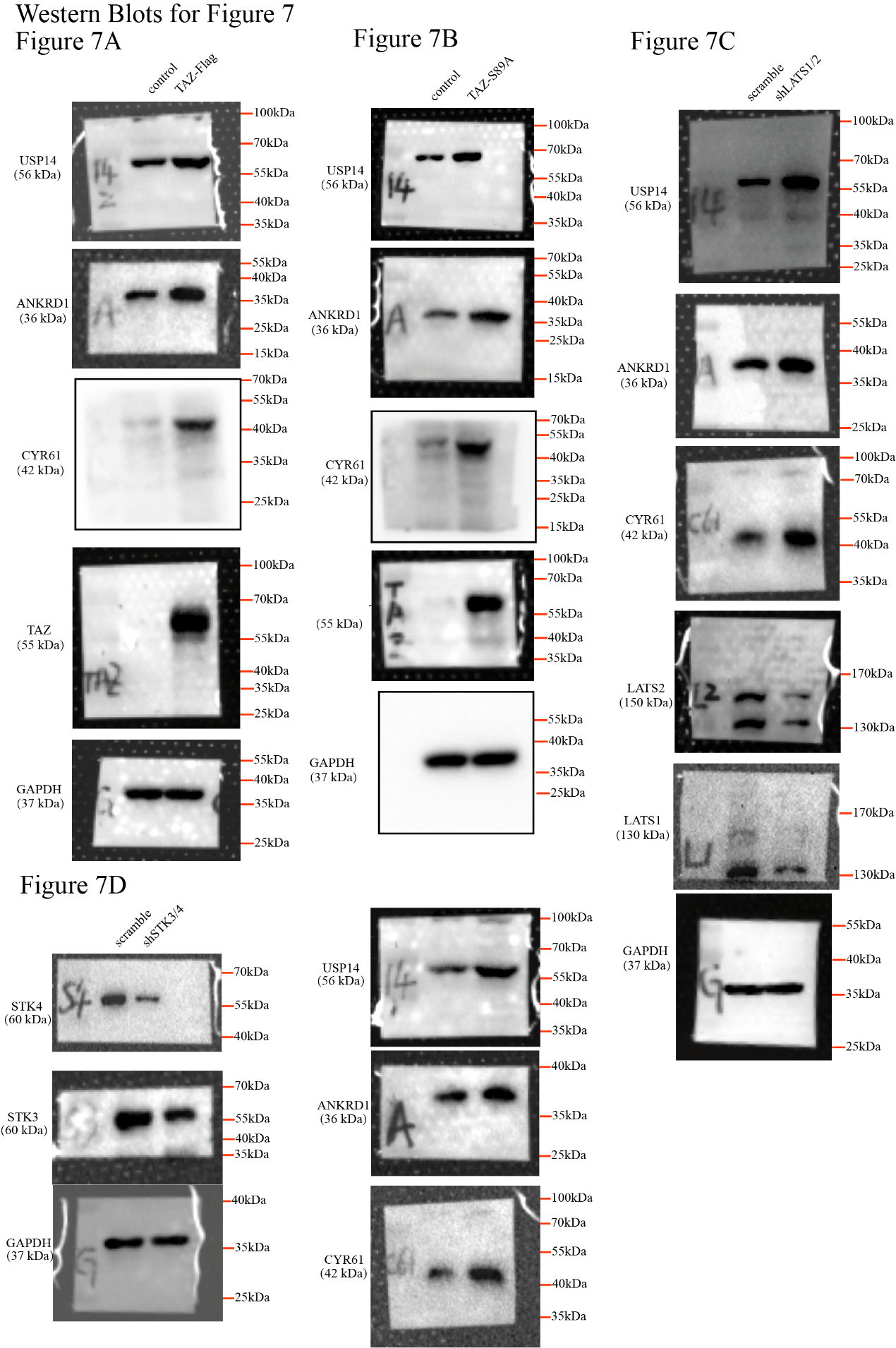


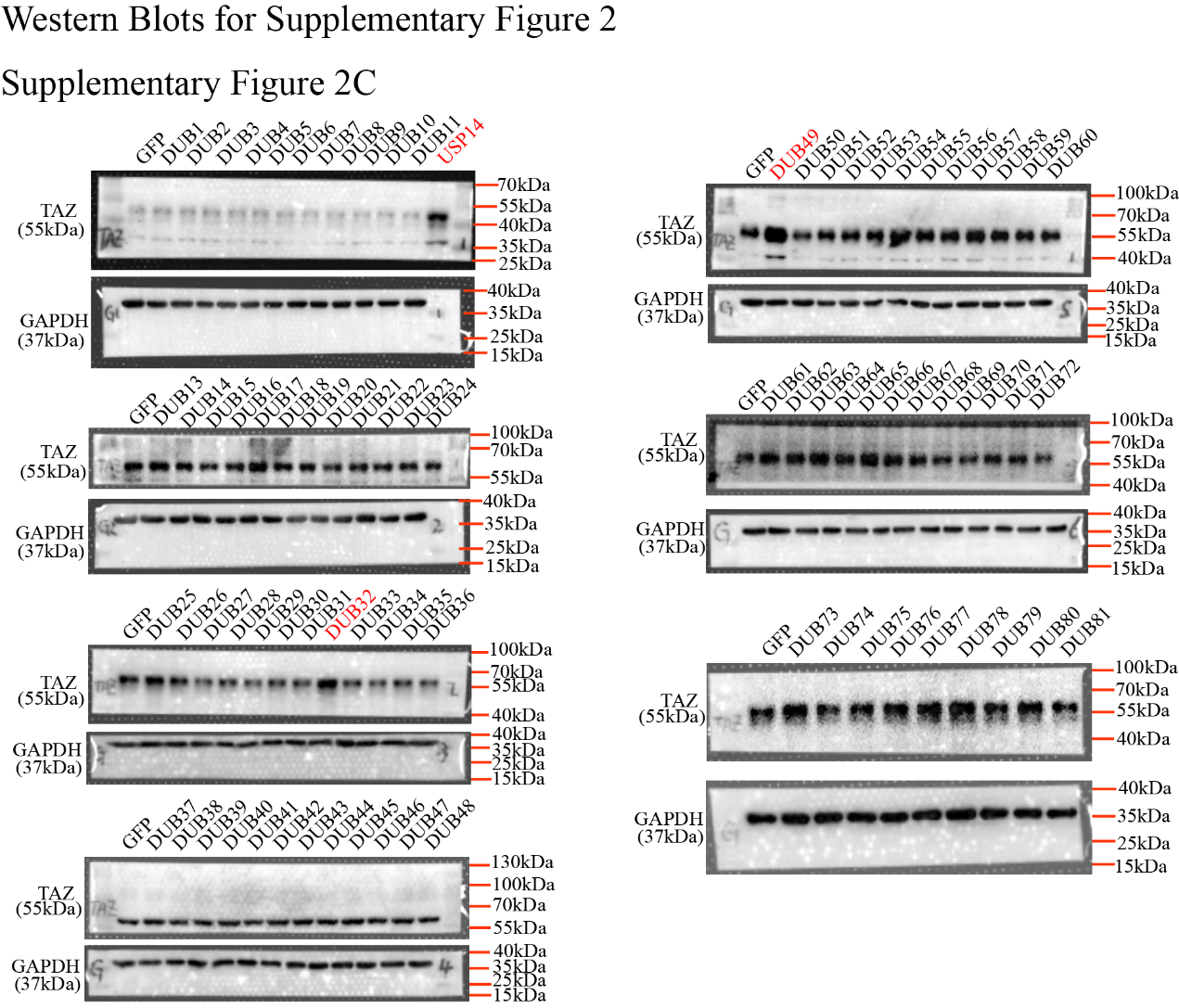


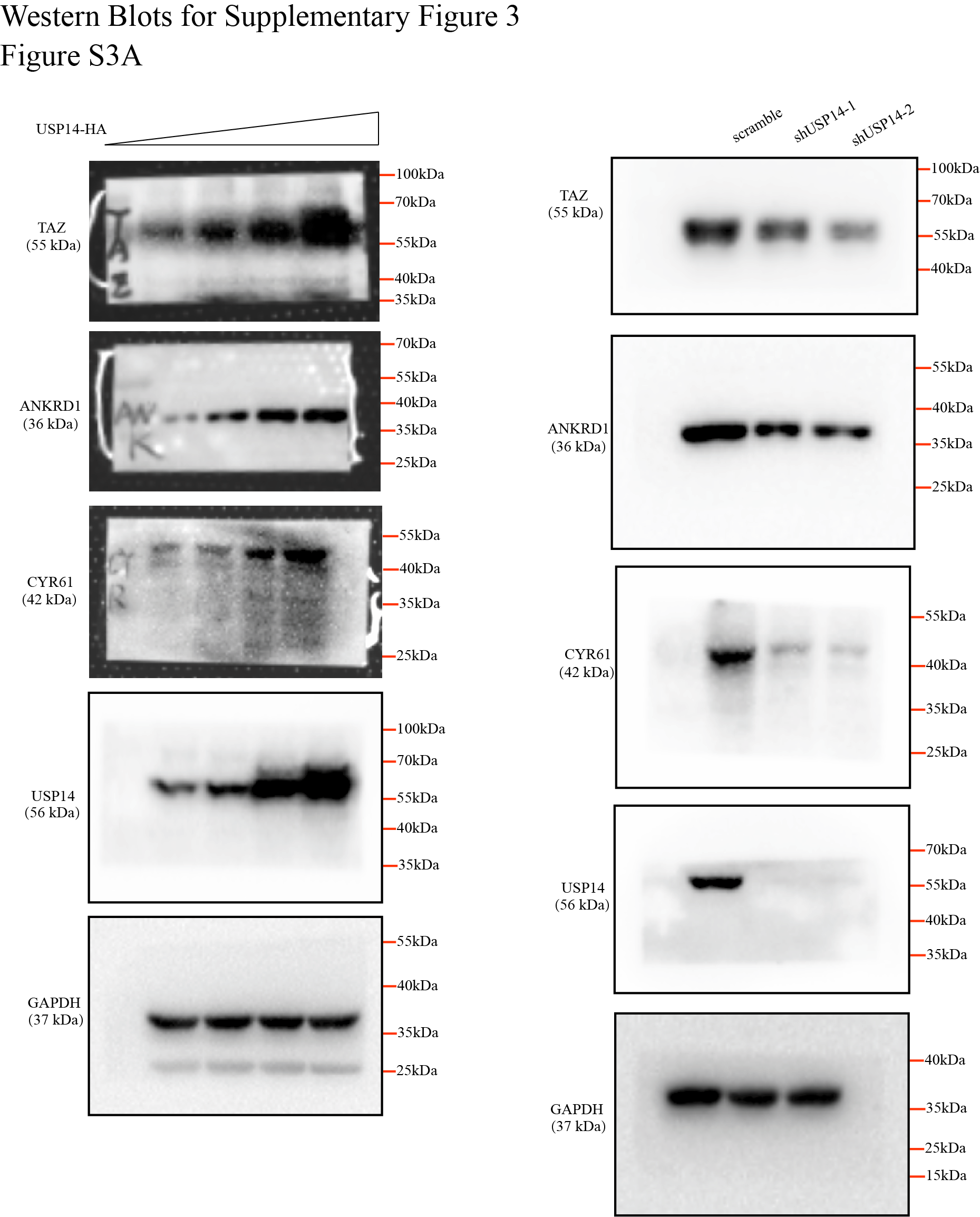


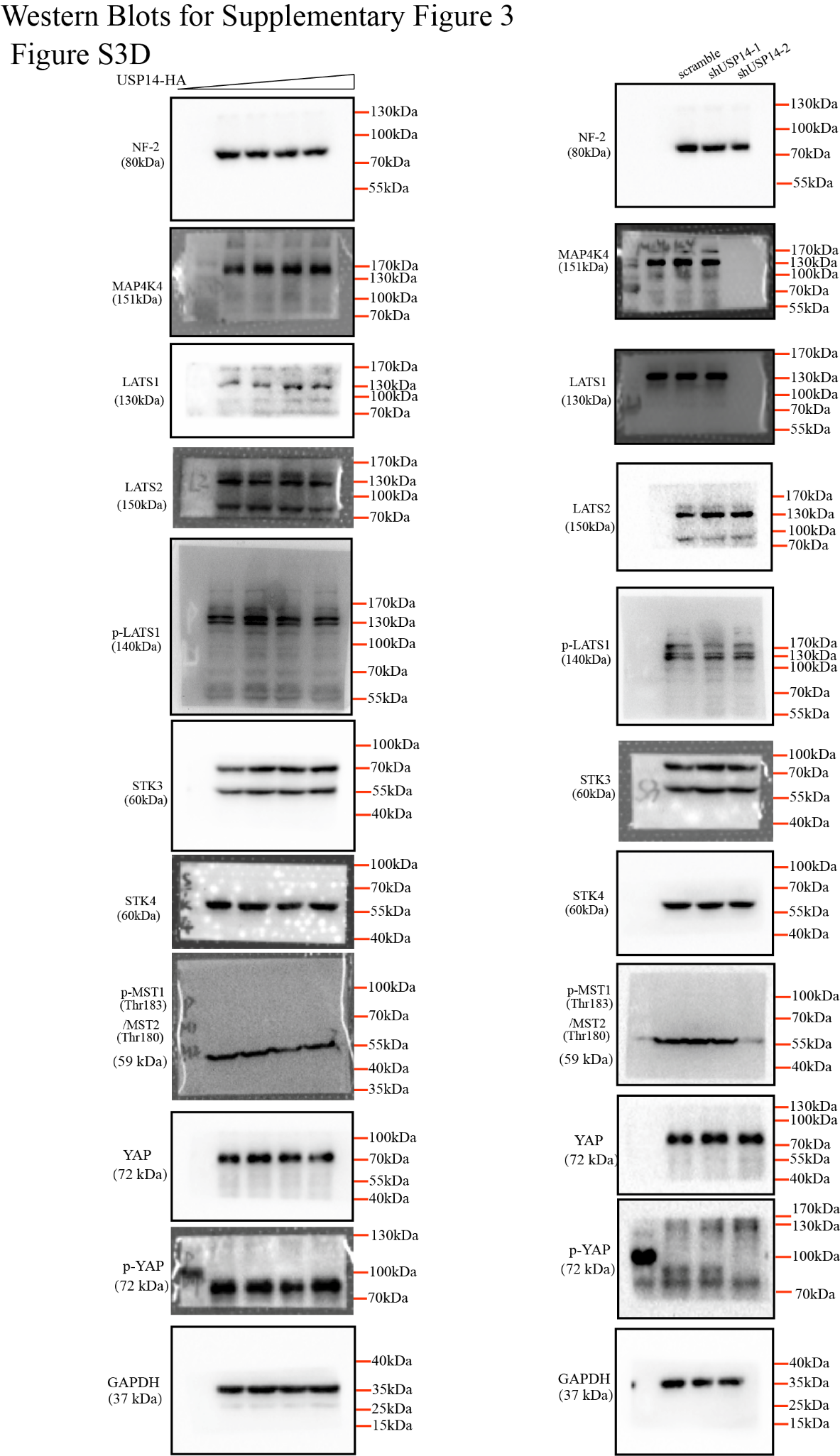


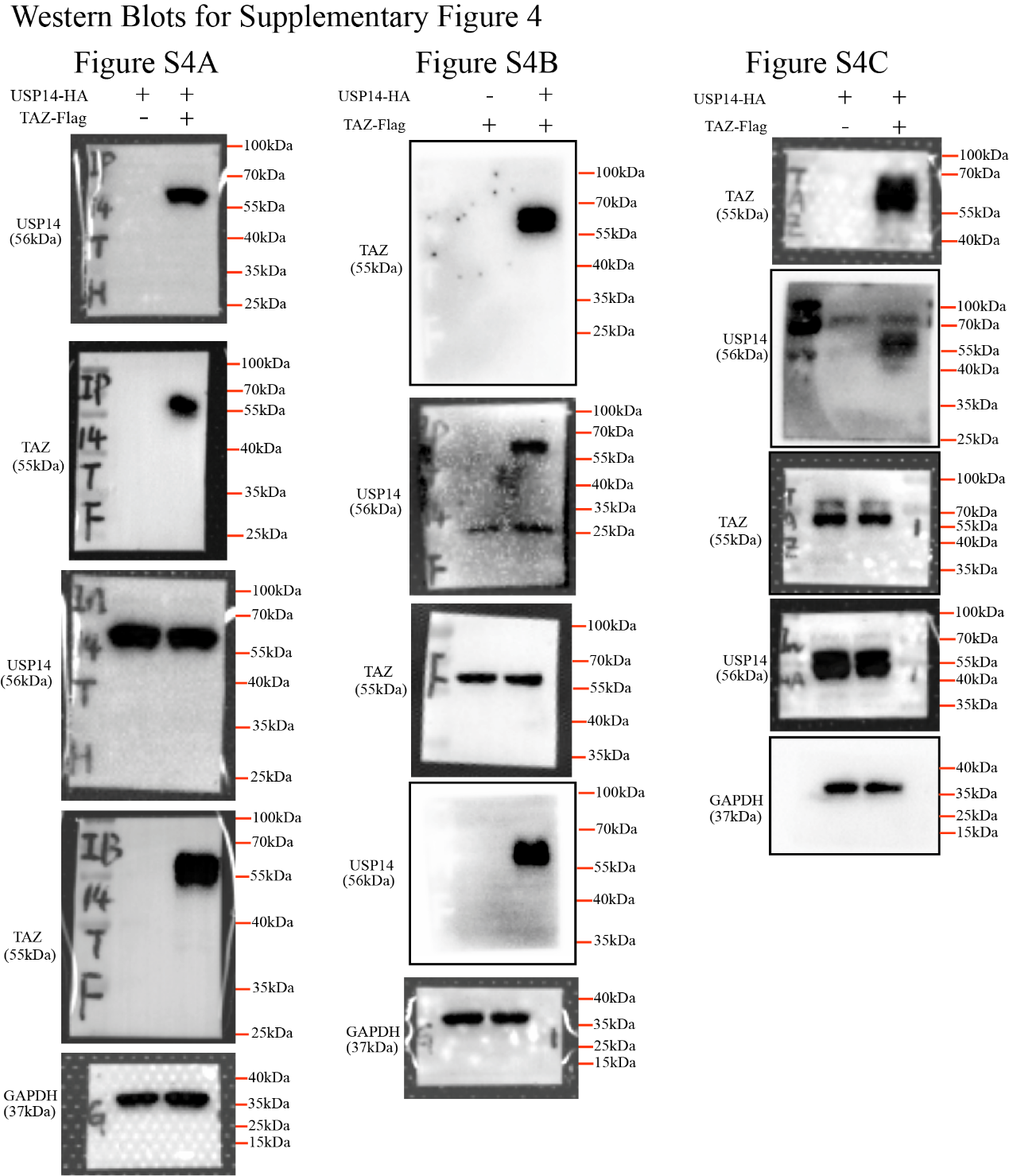


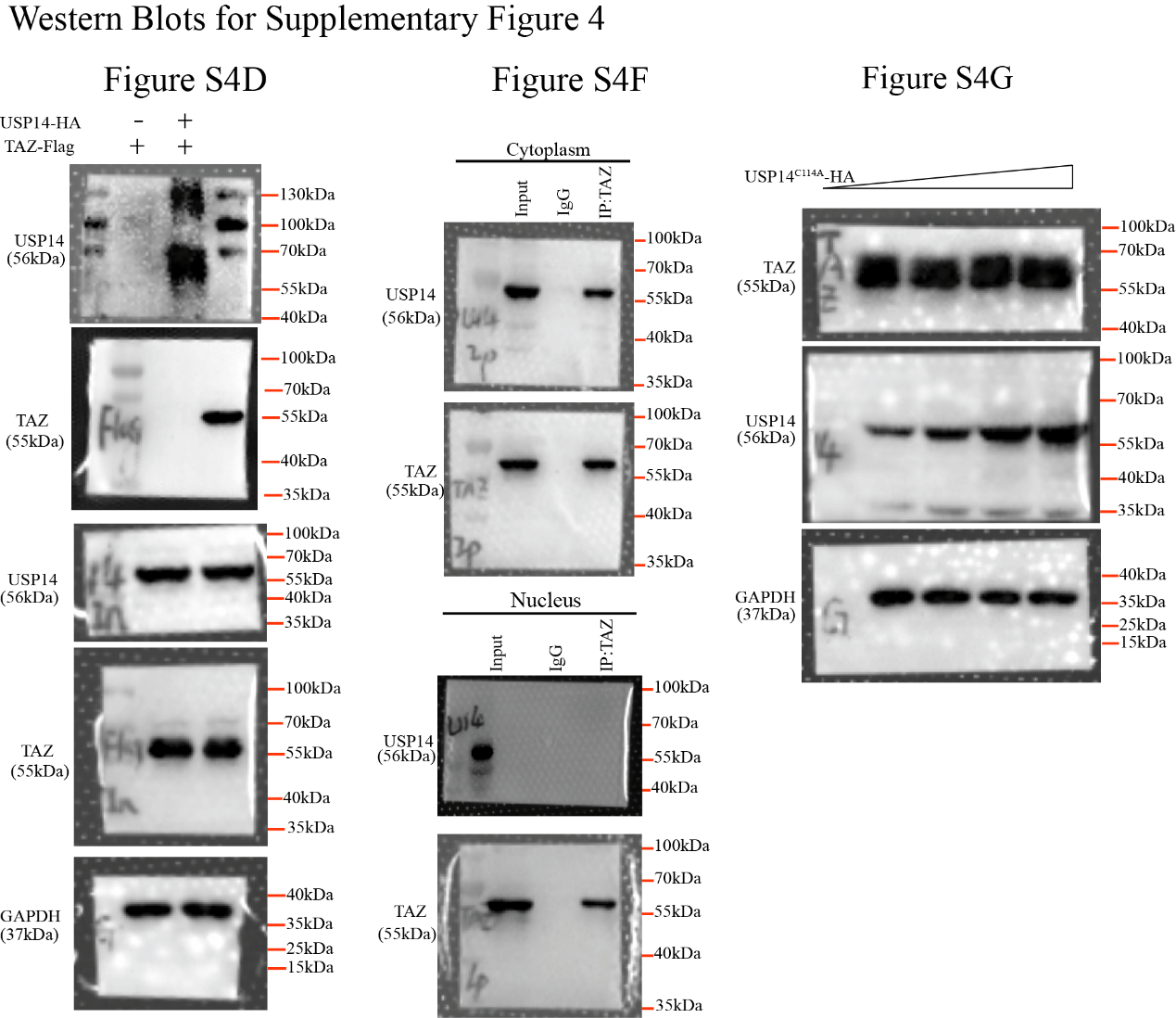


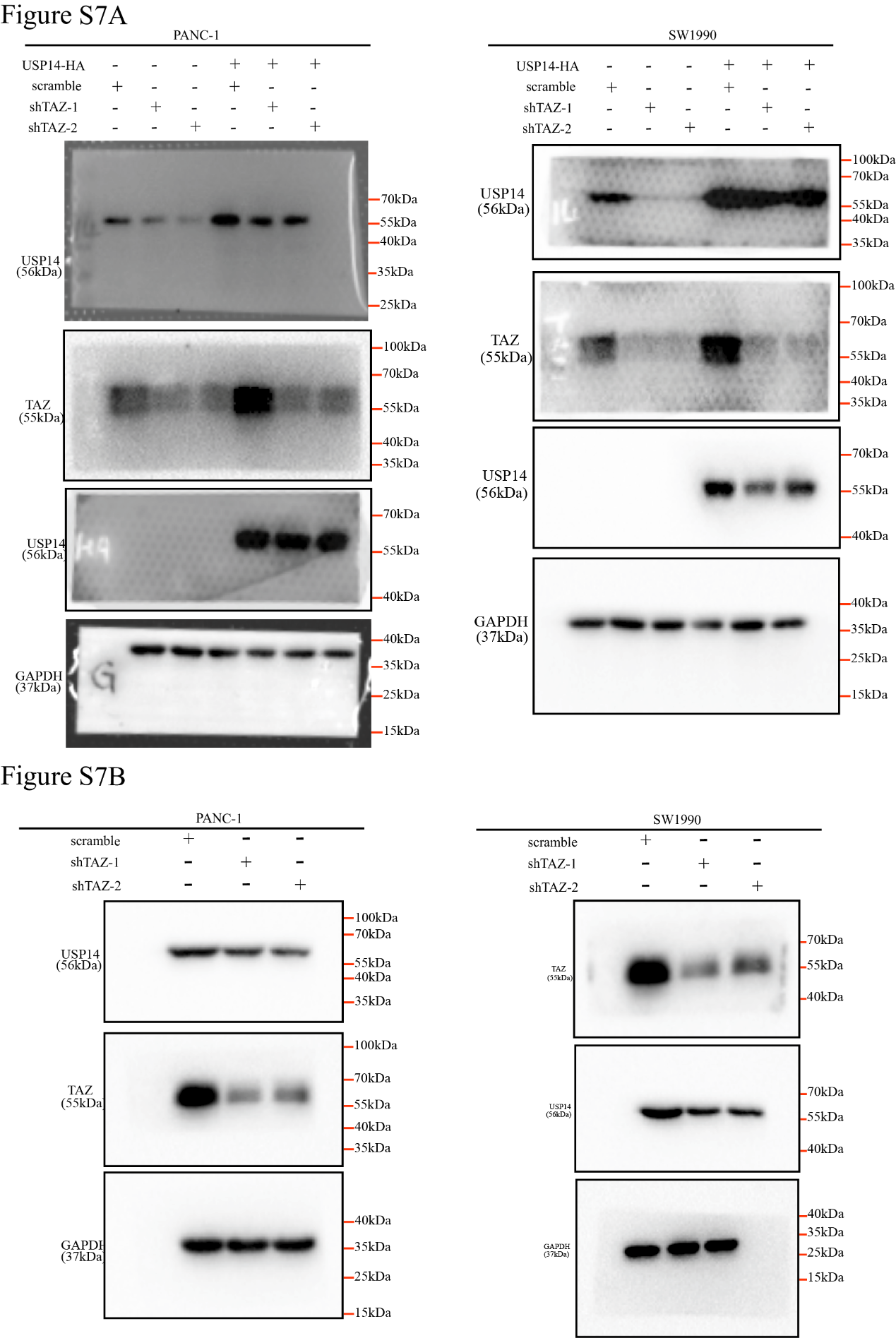


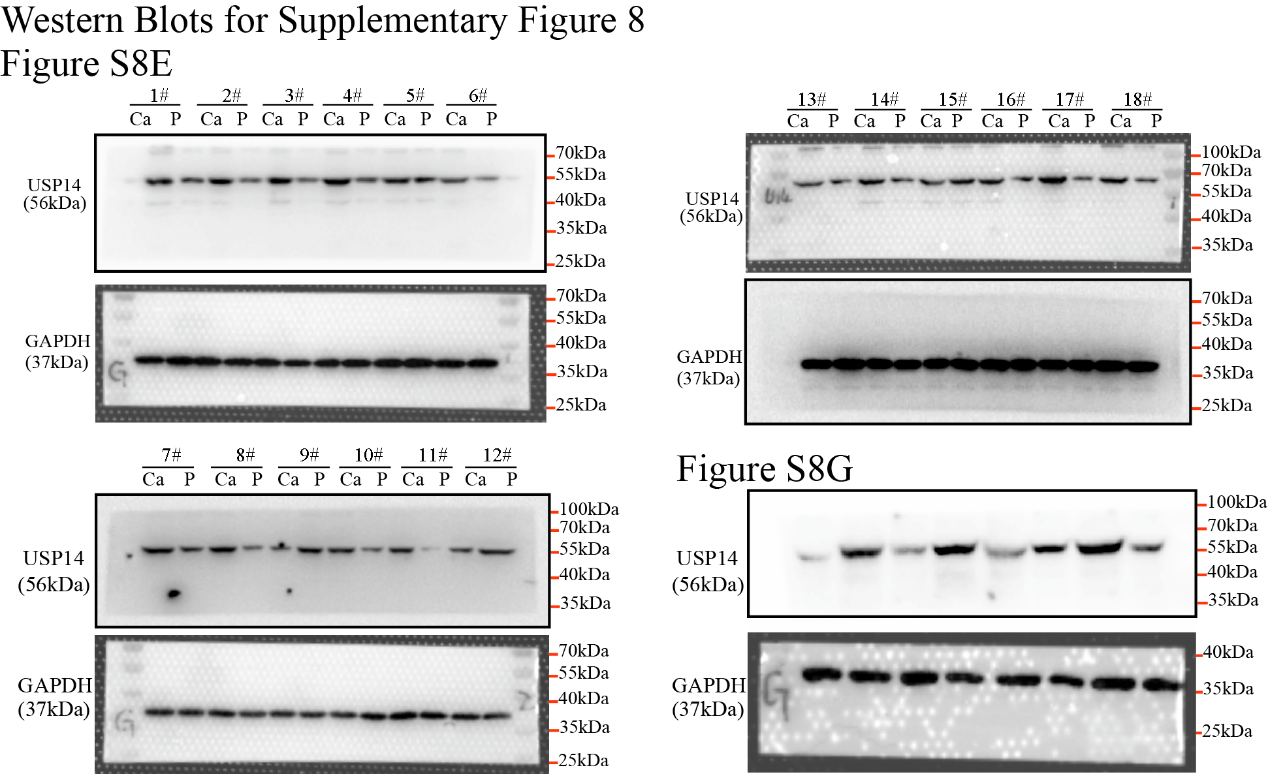


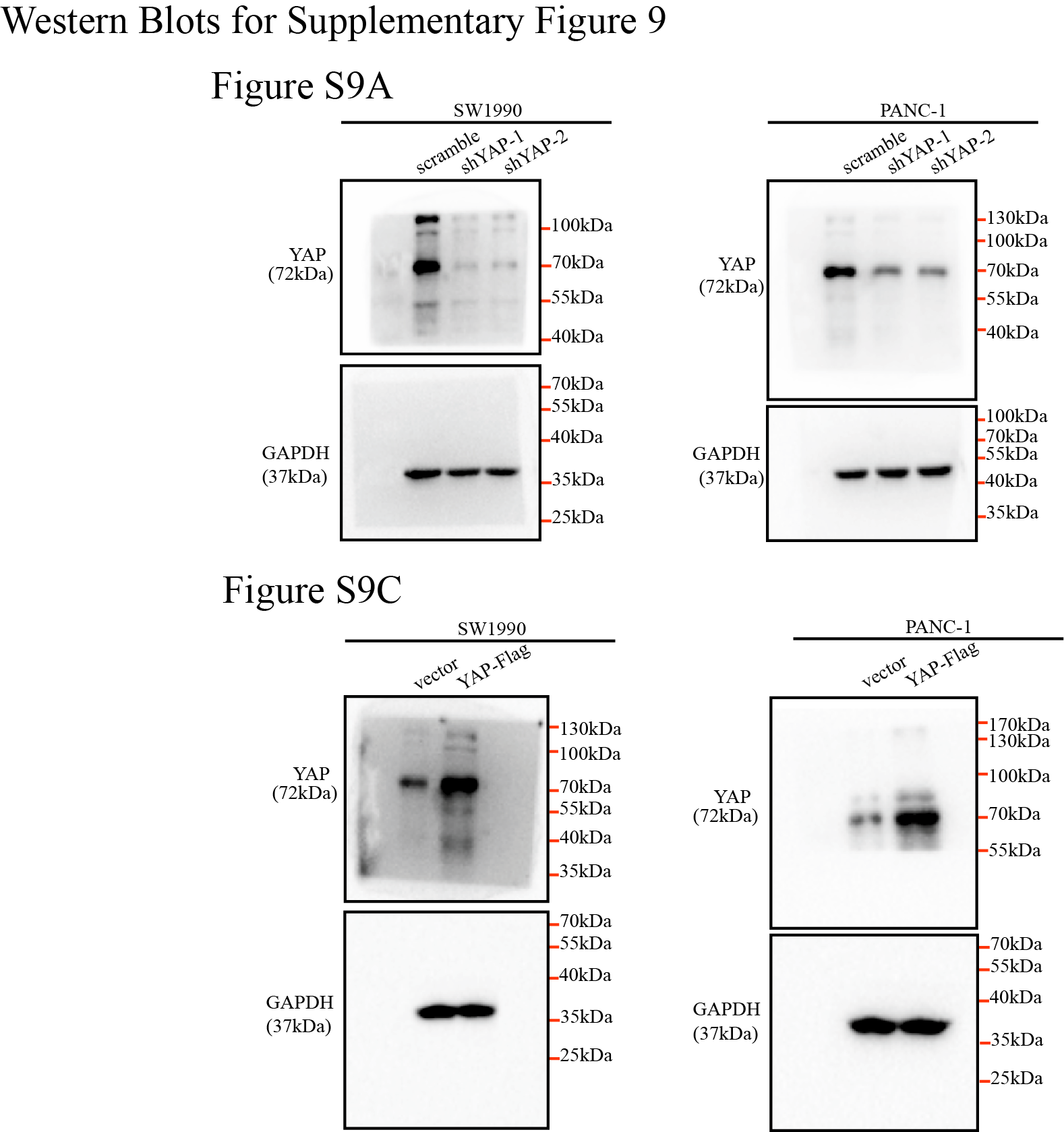

Supplement: Supplementary file 14 — Original western blots [file 41418_2022_1040_MOESM14_ESM.docx]
