# Supplementary material for: A self-amplifying USP14-TAZ loop drives the progression and liver metastasis of pancreatic ductal adenocarcinoma
Source: Cell Death Differ. 2022 Jul 29;30(1):1–15. doi: 10.1038/s41418-022-01040-w (PMC9883464; doi:10.1038/s41418-022-01040-w)
Supplement: Supplementary file 11 — Supplementary Table [file 41418_2022_1040_MOESM11_ESM.docx]

**Table S1. Clinicopathological characteristics of patient samples and expression of USP14 in Pancreatic ductal adenocarcinoma**

|  | **Pancreatic ductal adenocarcinoma (n=88)** | |
| --- | --- | --- |
| **Characteristics** | **No. of cases** | **Percentage (%)** |
| **Age(y)** |  |  |
| < 60 | 46 | 52.3 |
| ≥ 60 | 42 | 47.7 |
| **Gender** |  |  |
| Male | 60 | 68.2 |
| Female | 28 | 31.8 |
| **Clinical stage** |  |  |
| IA-IB | 47 | 53.4 |
| IIA-III | 41 | 46.6 |
| **T classification** |  |  |
| T1 | 16 | 18.2 |
| T2-T3 | 72 | 81.8 |
| **N classification** |  |  |
| N0 | 54 | 61.4 |
| N1-N2 | 34 | 38.6 |
| **Pathologic differentiation** |  |  |
| well | 12 | 13.6 |
| moderately/poorly | 76 | 86.4 |
| **USP14 expression** |  |  |
| Low expression | 33 | 37.5 |
| High expression | 55 | 62.5 |
| **Survival state** |  |  |
| Live | 29 | 33 |
| Dead | 59 | 67 |

**Table S2. Correlation between USP14 expression and clinicopathologic characteristics of Pancreatic ductal adenocarcinoma patients**

|  | **USP14 expression** | |  | |
| --- | --- | --- | --- | --- |
| **Characteristics** | **Low expression,  No. of cases** | **High expression,  No. of cases** | | ***P* value** |
| **Age(y)** |  |  | |  |
| ＜60 | 17 | 29 | | 0.912 |
| ≥60 | 16 | 26 | |  |
| **Gender** |  |  | |  |
| Male | 23 | 37 | | 0.813 |
| Female | 10 | 18 | |  |
| **Clinical stage** |  |  | |  |
| IA-IB | 29 | 18 | | **<0.001** |
| IIA-III | 4 | 37 | |  |
| **T classification** |  |  | |  |
| T1 | 14 | 2 | | **0.001** |
| T2-T3 | 19 | 53 | |  |
| **N classification** |  |  | |  |
| N0 | 31 | 23 | | **<0.001** |
| N1-N2 | 2 | 32 | |  |
| **Pathologic differentiation** |  |  | |  |
| well | 4 | 8 | | 0.748 |
| moderately/poorly | 29 | 47 | |  |
| **Survival state** |  |  | |  |
| Live | 24 | 5 | | **<0.001** |
| Dead | 9 | 50 | |  |

**Table S3. Univariate analysis of various prognostic parameters in patients with Pancreatic ductal adenocarcinoma cox-regression analysis**

|  | **Univariate analysis** | | |
| --- | --- | --- | --- |
| **Characteristics** | ***P* value** | **Hazard ratio** | **95% confidence interval** |
| **Clinical stage (Ref=IA)** | <0.001 | 4.246 | 2.457-7.338 |
| **T classification (Ref=T1)** | 0.003 | 4.133 | 1.638-10.427 |
| **N classification (Ref=N0)** | <0.001 | 3.43 | 2.028-5.802 |
| **USP14 expression (Ref=Low)** | <0.001 | 6.53 | 3.063-13.923 |

**Table S4. Multivariate analysis of various prognostic parameters in patients with Pancreatic ductal adenocarcinoma cox-regression analysis**

|  | | **Multivariate analysis** | | |
| --- | --- | --- | --- | --- |
| **Characteristics** | ***P* value** | | **Hazard ratio** | **95% confidence interval** |
| **Clinical stage (Ref=IA)** | 0.002 | | 5.261 | 1.833-15.101 |
| **T classification (Ref=T1)** | 0.036 | | 2.918 | 1.073-7.937 |
| **USP14 expression (Ref=Low）** | 0.009 | | 3.225 | 1.332-7.804 |

**Table S5. Information for antibodies used in this study**

| **Protein** | **Assay** | **Catalog number** | **Company** |
| --- | --- | --- | --- |
| *GAPDH* | WB | 60004-1-Ig | Proteintech |
| *TAZ*  *TAZ*  *USP14*  *NF-2*  *LATS1*  *LATS2*  *STK3*  *STK4*  *YAP1*  *p-YAP1*  *CYR61*  *ANKRD1*  *KI67*  *Flag*  *HA* | WB, IHC  IF  WB, IHC, IF  WB  WB  WB  WB  WB  WB  WB  WB  WB  IHC  WB  WB | 83669  66500-1-Ig  A16643  A0739  A17992  A16249  A6992  A8043  A1002  AP0489  26689-1-AP  11427-1-AP  27309-1-AP  F7425  AE008 | Cell Signaling Technology  Proteintech  ABclonal  ABclonal  ABclonal  ABclonal  ABclonal  ABclonal  ABclonal  ABclonal  Proteintech  Proteintech  Proteintech  Sigma-Aldrich  ABclonal |

**Table S5. (continue)**

| **Protein** | **Assay** | **Catalog number** | **Company** |
| --- | --- | --- | --- |
| *MYC* | WB | 16286-1-AP | Proteintech |
| *P-LATS1 (Ser909)*  *P-MST1(Thr183)*  */MST2 (Thr180)*  *MAP4K4*  *ACTB* | WB  WB  WB  WB | 9157  80093-1-RR  55247-1-AP  AC026 | Cell Signaling Technology  Proteintech  Proteintech  ABclonal |

**Table S6. Primers for quantitative PCR**

| **Primer set** | **Primers** | **Sequence (5’-3’)** | **Product size (bp)** | | |
| --- | --- | --- | --- | --- | --- |
| *ACTB* | Forward  Reverse | 5'- CATGTACGTTGCTATCCAGGC -3'  5'- CTCCTTAATGTCACGCACGAT -3' | | 250 |  |
| *TAZ*  *USP14*  *YAP1*  *CYR61*  *ANKRD1* | Forward  Reverse  Forward  Reverse  Forward  Reverse  Forward  Reverse  Forward  Reverse | 5'- GTCCTACGACGTGACCGAC -3'  5'- CACGAGATTTGGCTGGGATAC-3'  5'- TGGCTTCAGCGCAGTATATTAC -3'  5'- CCTTGTTCACCTTTCTCGGCA -3'  5'- TAGCCCTGCGTAGCCAGTTA -3'  5'- TCATGCTTAGTCCACTGTCTGT -3'  5'- AGCCTCGCATCCTATACAACC -3'  5'- TTCTTTCACAAGGCGGCACTC -3'  5'- ATCCGACTCCTGATTATGTATGG -3'  5'- GCTATGCGAGAGGTCTTGTAG -3' | 223  142  177  143  149 | |  |

**Table S7. Targeting sequences for shRNAs**

| **shRNAs** | **Sequences** |
| --- | --- |
| sh*TAZ*#1 | 5'- GCGATGAATCAGCCTCTGAAT -3' |
| sh*TAZ*#2 | 5'- CCAGGAACAAACGTTGACTTA -3' |
| sh*USP14*#1 | 5'- CGCAGAGTTGAAATAATGGAA -3' |
| sh*USP14*#2 | 5'- CCCAAGATTCAGCAGTCAGAT -3' |
| *shYAP#1* | 5'- GCCACCAAGCTAGATAAAGAA -3' |
|  |  |
| *shYAP#2* | 5'- CAGGTGATACTATCAACCAAA -3' |
|  |  |
| sh*LATS1*#1 | 5'- GAAGATAAAGACACTAGGAAT -3' |
| sh*LATS2*#1 | 5'- CTACTCGCCATACGCCTTTAA -3' |
| sh*STK3*#1 | 5'- CCGGTCAAGTTGTCGCAATTA -3' |
| sh*STK4*#1 | 5'- GGACCTGCATCATGAACAATG -3' |

**Table S8. Primers for ChIP-qPCR**

| **Primer set** | **Primers** | **Sequence (5’-3’)** |
| --- | --- | --- |
| USP14-Region1 | Forward  Reverse | 5'- TATTTTGAGGCAGGGTCTCG -3'  5'- CCTGCGGCAGATGAAGTG -3' |
| USP14-Region2  USP14-Region3  CTGF | Forward  Reverse  Forward  Reverse  Forward  Reverse | 5'- TGTCAAATTCTGCTGCTGTTTT -3'  5'- TGGGCAAGGAGTCGCTTC -3'  5'- TGTTCAGCATTTTACCCACAGA -3'  5'- GCTGTGAACACTGTTGAATGACA -3'  5'- TGTGCCAGCTTTTTCAGACG -3'  5'- TGAGCTGAATGGAGTCCTACACA -3' |
