# Supplementary material for: A self-amplifying USP14-TAZ loop drives the progression and liver metastasis of pancreatic ductal adenocarcinoma
Source: Cell Death Differ. 2022 Jul 29;30(1):1–15. doi: 10.1038/s41418-022-01040-w (PMC9883464; doi:10.1038/s41418-022-01040-w)
Supplement: Supplementary file 12 — Author Contribution form [file 41418_2022_1040_MOESM12_ESM.pdf]

DECLARATION OF CONTRIBUTIONS TO ARTICLE

Manuscript Number: CDD-21-2074

Journal Name: *Cell Death & Differentiation* (the 'Journal')

Proposed Title of the Contribution: Author contributions (the 'Contribution)

Author(s): Chunle Zhao, Jun Gong, Yu Bai, Taoyuan Yin, Min Zhou, Shutao Pan, Yuhui Liu, Yang Gao, Zhenxiong Zhang, Yongkang Shi, Feng Zhu, Hang Zhang, Min Wang, Renyi Qin (the 'Authors')

For all *CDD* articles, each person named as an author in the published version must be able to show he or she has contributed substantially to the article.

Authorship credit should be based on 1) substantial contributions to conception and design, acquisition of data, or analysis and interpretation of data; 2) drafting the article or revising it critically for important intellectual content; and 3) final approval of the version to be published. Authors should meet conditions 1, 2 and 3.

Any person who cannot be shown to have made a substantial contribution to the article cannot be listed as an author in the final version. The name of any person who is deemed to have made a minor contribution can, however, appear in the Acknowledgments section of the article.

Please complete the table below to indicate the contributions of all named authors to the manuscript.

Author Full Name:	Specification of Contribution to the Manuscript:
Chunle Zhao, Jun Gong, Feng Zhu, Hang Zhang, Min Wang, Renyi Qin	Participate in research design
Chunle Zhao, Jun Gong	Draft the paper.
Hang Zhang, Min Wang, Renyi Qin	Supervise the study.
hunle Zhao, Jun Gong, Shutao Pan, Yuhui Liu, Yang Gao	Constructe plasmid and culture cells.
Chunle Zhao, Jun Gong, Yu Bai	Performe co-IP assay, GST pull down, CHIP and luciferase assay.
Chunle Zhao, Jun Gong, Yu Bai, Taoyuan Yin, Min Zhou	Collecte human sample and performed WB and PCR.
Chunle Zhao, Jun Gong, Feng Zhu,	Put forward constructive suggestions
Chunle Zhao, Jun Gong, Zhenxiong Zhang, Yongkang Shi	Feed and monitor mice.
Taoyuan Yin, Min Zhou, Shutao Pan, Yuhui Liu, Yang Gao, Zhenxiong Zhang	Analyse the data.
Jun Gong, Feng Zhu,Min Wang, Renyi Qin	Fund the project.

Please complete the table below to indicate the contributions of all named authors to the figures.

Figure 1:
Chunle Zhao, Jun Gong, Shutao Pan, Yuhui Liu, Yang Gao, Hang Zhang, Min Wang, Renyi Qin

Figure 2:
Chunle Zhao, Jun Gong, Yu Bai, Taoyuan Yin, Min Zhou, Hang Zhang, Min Wang, Renyi Qin

Figure 3:
Chunle Zhao, Jun Gong, Yu Bai, Taoyuan Yin, Min Zhou, Feng Zhu, Hang Zhang, Min Wang, Renyi Qin

Figure 4:
Chunle Zhao, Jun Gong, Yuhui Liu, Yang Gao, Shutao Pan,Min Wang, Renyi Qin

Figure 5:
Chunle Zhao, Jun Gong, Shutao Pan, Yuhui Liu, Yang Gao ,Hang Zhang, Min Wang, Renyi Qin

Figure 6:
Chunle Zhao, Jun Gong, Zhenxiong Zhang, Yongkang Shi, Hang Zhang, Min Wang, Renyi Qin

Figure7:Chunle Zhao, Jun Gong, Yu Bai,Feng Zhu,Hang Zhang, Min Wang, Renyi Qin

Figure8:Chunle Zhao, Jun Gong, Zhenxiong Zhang, Yongkang Shi, Min Wang, Renyi Qin

Signed for and on behalf of the Author(s):	Print Name:	Date:
Renyi Qin	Renyi Qin	April 6, 2022
